# The RNase MCPIP3 promotes skin inflammation by orchestrating myeloid cytokine response

**DOI:** 10.1038/s41467-021-24352-w

**Published:** 2021-07-02

**Authors:** Bo Liu, Jiancheng Huang, Amina Ashraf, Oindrila Rahaman, Jing Lou, Ling Wang, Peiliang Cai, Jinping Wen, Shoaib Anwaar, Xiaoli Liu, Hai Ni, Dipyaman Ganguly, Jijun Zhao, Cliff Y. Yang

**Affiliations:** 1 Department of Immunology, Sun Yat-sen University, Zhongshan School of Medicine, Guangzhou, Guangdong, China; 2 IICB-Translational Research Unit of Excellence, CSIR-Indian Institute of Chemical Biology, Kolkata, India; 3 Department of Rheumatology and Immunology, The First Affiliated Hospital, Sun Yat-sen University, Guangzhou, China; 4 Key Laboratory of Tropical Disease Control (Sun Yat-Sen University), Ministry of Education, Guangzhou, China

**Keywords:** Tumour-necrosis factors, Epigenetics in immune cells, Plasmacytoid dendritic cells, Monocytes and macrophages, Psoriasis

## Abstract

CCCH zinc finger proteins resolve immune responses by degrading the mRNAs of inflammatory cytokines such as tumor necrosis factor (TNF) and interleukin (IL)-6. Here we report that one such family member, monocyte chemotactic protein-induced protein 3 (MCPIP3, also named ZC3H12C or Regnase-3), promotes skin inflammation by simultaneously enhancing TNF in macrophages and repressing IL-6 in plasmacytoid dendritic cells (pDCs). MCPIP3 is positively associated with psoriasis pathogenesis, and highly expressed by macrophages and pDCs. MCPIP3-deficient macrophages produce less TNF and IL-12p40. However, MCPIP3-deficient pDCs secrete significantly more IL-6. This enhanced intradermal IL-6 may alleviate imiquimod-induced skin inflammation. As a result, MCPIP3-deficient mice are protected from imiquimod-induced psoriasiform lesions. Furthermore, early exposure to pDC-derived IL-6 suppresses macrophage-derived TNF and IL-12p40. Mechanistically, MCPIP3 could directly degrade mRNAs of IL-6, Regnase-1, and IκBζ. In turn, Regnase-1 could degrade MCPIP3 mRNAs. Our study identifies a critical post-transcriptional mechanism that synchronizes myeloid cytokine secretion to initiate autoimmune skin inflammation.

## Abstract (web summary)

Zinc finger proteins are involved in the resolution of immune responses and function by degrading mRNA of inflammatory cytokines. Here the authors show MCPIP3 promotes skin inflammation via modification of cytokine profiles in pDCs and macrophages.

## Introduction

A delicate balance between pro-inflammatory and anti-inflammatory immune responses is required to clear pathogens without causing autoimmunity^1^. Tumor necrosis factor (TNF) and interleukin (IL)-6 are potent inflammatory cytokines that regulate immunity and disease pathogenesis^2^. Aberrant overproduction of TNF had been implicated in the pathogenesis of chronic inflammatory diseases such as rheumatic arthritis, psoriatic lesions/arthritis, and Crohn’s disease^3,4^. Similarly, elevated IL-6 expression led to idiopathic juvenile arthritis and other chronic inflammations^2,5^. While TNF-targeted blockade had been shown to be effective in treating psoriasis and other autoimmune diseases, IL-6-targeted blockade had not been equally successful^6^. Whereas the transcriptional control of TNF and IL-6 has been extensively investigated, the post-transcriptional regulation of TNF/IL-6 is less understood.

RNA-binding proteins are major components of regulatory feedback loops that maintain immune tolerance and limit inflammation^7^. Cys-Cys-Cys-His (CCCH) zinc finger proteins are a subclass of RNA-binding proteins that regulate mRNA splicing, polyadenylation, export, translation, and decay^8^. Although the physiological functions of most CCCH zinc finger proteins are still uncertain, recent reports suggest that the tristetraprolin (TTP), roquin, and monocyte chemotactic protein-induced protein (MCPIP) families, are critical negative regulators of inflammatory responses^9–11^. TTP, roquin 1, and Regnase-1 (also known as MCPIP1; encoded by *Zc3h12a*) belong to an intricate mRNA degradation network that maintains immune homeostasis by repressing inflammatory cytokines such as IL-6 and TNF in macrophages^12–15^. Besides macrophages, roquin 1 and Regnase-1 suppress the activation, differentiation, and IL-17A production of CD4^+^ helper T cells^16–20^. TTP, roquin 1 and Regnase-1 could be evolutionarily closely related, as their genetically deficient mouse models suffer from a multitude of severe inflammatory autoimmune diseases^10,15,21,22^. By comparison, other MCPIP members appear to have similar but less significant roles than Regnase-1. MCPIP4 (also named TFL; encoded by *Zc3h12d*)-deficient mice were healthy but exhibited excessive T-cell activation in a multiple sclerosis model^23^. MCPIP3 (also named Regnase-3; encoded by *Zc3h12c*)-deficient mice were also healthy, despite some lymphadenopathy and an increase in serum interferon (IFN)-γ^24^. Thus far, all characterized CCCH zinc finger proteins have been reported to be repressors of cytokine production and immune cell activation.

In humans, single nucleotide polymorphisms (SNPs) in MCPIP3 were identified in genome-wide association studies (GWAS) to be highly associated with psoriasis^25^. Psoriasis is a common chronic inflammatory skin disease with a prevalence of an estimated 1–3% of the worldwide population^26^. Heavy dermal infiltration of immune cells, such as myeloid cells and T cells, is critical for epidermal thickening^27^. Subsequently, TNF and IL-23 produced by myeloid cells drive the production of IL-17A by T cells^28^. Clinical studies and targeted therapies have demonstrated that the cytokines TNF, IL-23, and IL-17A have pivotal roles in psoriasis pathogenesis^29^. Thus, the exact role of MCPIP3 in psoriasis warrants further investigation.

In this report, we identify MCPIP3 as a critical regulator of inflammatory cytokines during early phase of psoriasis pathogenesis. We show that MCPIP3 promotes TNF production in macrophages, and represses IL-6 secretion in pDCs. We demonstrate that MCPIP3 degrades multiple targets, such as *Il6, Zc3h12a*, and *Nfkbiz* mRNAs, to achieve this differential cytokine regulation.

## Results

### MCPIP3 is positively associated with psoriasis pathogenesis

**Fig. 1:**
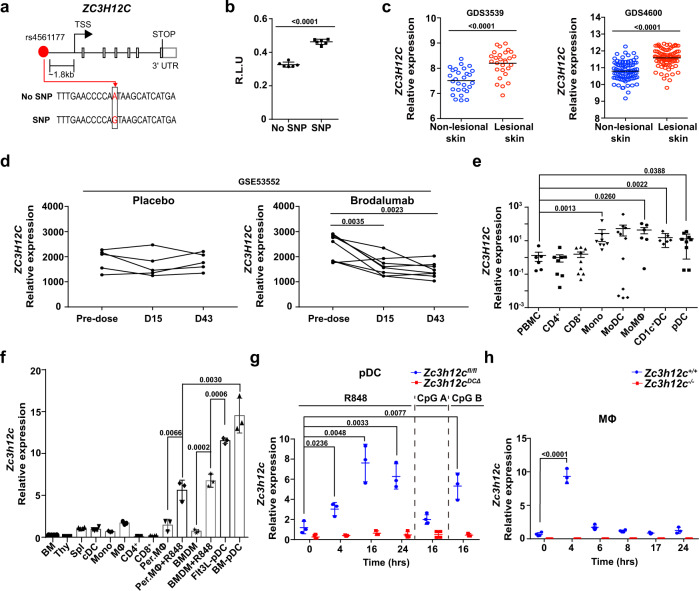
MCPIP3 is positively associated with psoriasis pathogenesis. **a** Schematics indicating the location of SNP rs4561177 in human *ZC3H12C*. **b** Regulation of *ZC3H12C* expression by SNP rs4561177 was measured by a luciferase reporter system. The promoter region (~1 kb) containing the SNP was inserted upstream of the luciferase gene, and then transfected into 293 T cells for assessment of reporter activity (R.L.U) (*n* = 6 replicates). **c** *ZC3H12C* expression in psoriatic lesion (red) and non-lesions biopsies (blue) from psoriasis patients were analyzed (*n* = 28 individuals in GDS3539; *n* = 85 individuals in GDS4600). **d** *ZC3H12C* expression in psoriatic lesions after brodalumab treatment (GEO: GSE53552) was analyzed (*n* = 5 individuals for placebo, 8 individuals for brodalumab). **e** *ZC3H12C* expression in human peripheral blood mononuclear cells (PBMC) immune populations was measured by qPCR (*n* = 6 individuals for PMBC; *n* = 11 individuals for other groups). **f** *Zc3h12c* expression in murine WT immune populations was measured by qPCR (*n* = 3 mice per group). **g** *Zc3h12c* expression in sorted *Zc3h12c* ^fl/fl^ (blue) /*Zc3h12c* ^DCΔ^ (red) BM plasmacytoid dendritic cells (pDCs) was measured by qPCR after stimulation (*n* = 3 mice per group). **h** *Zc3h12c* expression in R848-treated *Zc3h12c*^+/+^ (blue)/*Zc3h12c*^−/−^(red) bone marrow-derived macrophages (BMDMs) was measured by qPCR at indicated timepoints (*n* = 3 mice per group). Data are representative at least three independent experiments (mean ± S.D.; two-tailed Student’s *t* test for murine samples; two-tailed Wilcoxon matched-pair signed rank test for human samples). Source data are provided as a  Source Data file.

A previously published GWAS study identified one SNP in *ZC3H12C* (the gene encoding MCPIP3) that is highly associated with psoriasis^25^. This SNP (rs4561177) is located at ~1.8 kb upstream from the *ZC3H12C* transcriptional start site (Fig. 1a). The bioinformatical database Genotype-Tissue Expression (GTEx, www.gtexportal.org) suggested that this particular SNP could be an expression quantitative trait locus (eQTL) that controls human gene transcription (Supplementary Fig. 1a). To see if this SNP affects *ZC3H12C* expression, we cloned ~1 kb of upstream promoter region including the SNP into a luciferase reporter system. We found that this single A to G conversion increased luciferase reporter expression by ~50% when compared to the same promoter region with no SNP (Fig. 1b). In order to determine if this increased MCPIP3 expression is correlated with psoriasis, we analyzed human *ZC3H12C* transcripts from psoriasis patient biopsies (GEO: GDS3539; GDS4600) (Fig. 1a)^30,31^. We found that *ZC3H12C* transcripts were significantly higher in lesional skin than non-lesional skin from the same psoriasis patients (Fig. 1c). Consistently, we found that *ZC3H12C* expression is normalized after successful clinical treatment of brodalumab, an anti-IL-17A neutralizing antibody (GEO: GSE53552) (Fig. 1d)^32^. Here, we showed that MCPIP3 expression is positively associated with psoriasiform lesions.

Next, we examined MCPIP3 mRNA transcripts in human and murine immune cells. From human peripheral blood mononuclear cells (PBMCs), we observed that *ZC3H12C* mRNA was highly expressed in a broad group of myeloid cells, including pDCs, CD1C^+^ DCs, monocytes and monocyte-derived macrophages (MoMΦs) (Fig. 1e). Human and murine MCPIP3 are highly conserved, especially at the key functional NYN nuclease and CCCH RNA-binding domains (100% conserved at the protein level) (Supplementary Fig. 1b). In naïve murine immune populations, *Zc3h12c* expression was the highest in plasmacytoid dendritic cells (pDCs) (Fig. 1f). This pattern is consistent with RNA-Seq data from the Immunological Genome Project (IMMGEN, www.immgen.org) (Supplementary Fig. 1c). In pDCs, *Zc3h12c* mRNA expression was further increased after TLR7/8 agonist R848 stimulation, with a peak around 16 h (Fig. 1g). Interestingly, *Zc3h12c* expression in pDCs was unchanged after TLR9 agonist CpG A activation (Fig. 1g). In contrast to CpG A, CpG B activates TLR9 in the late endosome and upregulates NFkB dependent cytokines IL-6 and TNF much more than CpG A^33^. We found that CpG B could upregulate *Zc3h12c* expression in pDCs, albeit to a lesser extent than R848 (Fig. 1g). Here we show that MCPIP3 expression is enriched in naïve and activated pDCs.

Unstimulated murine macrophages expressed relatively few MCPIP3 transcripts (Fig. 1f). After R848-activation, *Zc3h12c* transcripts was upregulated by >6-fold in bone marrow-derived CD11b^+^ F4/80^+^ macrophages (BMDMs) and by >3-fold in peritoneal cavity CD11b^+^ F4/80^+^ macrophages (Per. MΦs) (Fig. 1f). Still, *Zc3h12c* mRNA in unstimulated pDCs was ~1-fold higher than that of R848-activated macrophages (Fig. 1f). This meant that *Zc3h12c* expression in R848-activated pDCs was ~14-fold higher than that of activated macrophages (Fig. 1g). Next, we carefully monitored the kinetics of MCPIP3 expression in activated macrophages. *Zc3h12c* mRNA expression peaked at 4 h after R848 activation, but remained quite low at later timepoints (Fig. 1h). Here we show that MCPIP3 is transiently expressed in early activated macrophages.

To study the function of MCPIP3 in pDCs and macrophages, we generated germline genetic knockout mice (*Zc3h12c*^−^^/−^) and conditional knockout mice (*Zc3h12c*^fl/fl^) with *Lyz2-Cre* (*Zc3h12c*^MΦΔ^) and *Itgax-Cre* (*Zc3h12c*^DCΔ^) (Supplementary Fig. 1d–i)^34,35^. We observed low occurrence of lymphadenopathy in >12 weeks old *Zc3h12c*^−/−^ and *Zc3h12c*^MΦΔ^ mice, albeit this difference was not statistically significant (Supplementary Fig. 1j). The penetrance and severity of lymphadenopathy in our MCPIP3-deficient mice was much lower than previously reported^24^. For all our in vivo experiments, we utilized only 6–10 weeks old mice, which exhibited no signs of lymphadenopathy. In general, *Zc3h12c*-deficient mice were born at normal Mendelian ratios, had normal immune development, and appeared healthy for at least 1.5 years (Supplementary Fig. 2a). Most importantly, macrophage and DC populations in *Zc3h12c*^−/−^/*Zc3h12c*^MΦΔ^/*Zc3h12c*^DCΔ^ mice are normal compared to littermate controls (Supplementary Fig. 2b–i). Thus, we conclude that MCPIP3 is dispensable for macrophage or DC development.

### Early TNF production is impaired in MCPIP3-deficient macrophages

**Fig. 2:**
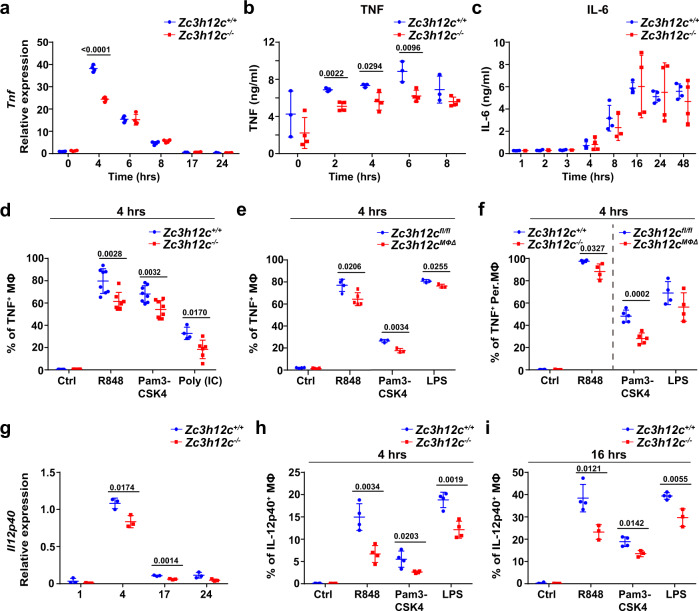
Early TNF production is impaired in MCPIP3-deficient macrophages. **a** *Tnf* expression in R848-treated *Zc3h12c*^+/+^(blue)/*Zc3h12c*^−/−^ (red) bone marrow-derived macrophages (BMDMs) was measured by qPCR at indicated timepoints (*n* = 3 mice per group). **b**, **c** TNF(b) and IL-6(c) secretion by R848-treated *Zc3h12c*^+/+^/*Zc3h12c*^−/−^ BMDMs was measured by ELISA at indicated timepoints (*n* = 3 mice for *Zc3h12c*^+/+^; *n* = 4 mice for *Zc3h12c*^−/−^). **d–f** TNF production by *Zc3h12c*^+/+^/*Zc3h12c*^−/−^ BMDMs (**d**, *n* = 7 mice for *Zc3h12c*^+/+^; *n* = 8 mice for *Zc3h12c*^−/−^), *Zc3h12c*^fl/fl^/*Zc3h12c*^MΦΔ^ BMDMs (**e**, *n* = 3 mice for *Zc3h12c*^fl/fl^; *n* = 5 mice for *Zc3h12c*^MΦΔ^), and purified *Zc3h12c*^+/+^/*Zc3h12c*^−/−^ or *Zc3h12c*^fl/fl^/*Zc3h12c*^MΦΔ^ peritoneal macrophages (Per.MΦ) (**f**, *n* = 3 mice for *Zc3h12c*^fl/fl^; *n* = 5 mice for *Zc3h12c*^MΦΔ^) was measured by intracellular staining after 4 h of stimulation with indicated ligands. **g** *Il12p40* expression in R848-treated *Zc3h12c*^+/+^/*Zc3h12c*^−/−^ BMDMs was measured by qPCR at various timepoints (*n* = 3 mice per group). **h**, **i** IL-12p40 production by *Zc3h12c*^+/+^/*Zc3h12c*^−/−^ BMDMs was measured by intracellular staining after 4 h (**h**, *n* = 4 mice per group) or 16 h (**I**, *n* = 4 mice for *Zc3h12c*^+/+^; *n* = 3 mice for *Zc3h12c*^−/−)^ stimulation with indicated ligands. Data are representative of eight (**d**, **e**), four (**a**, **b**), three (**h**, **i**), two (**c**, **f**, **g**) independent experiments (mean ± S.D.; two-tailed Student’s *t* test). Source data are provided as a Source Data file.

Since MCPIP3 was highly expressed in activated macrophages, we investigated the role of MCPIP3 in macrophage function. We found a significant reduction of *Tnf* transcripts or TNF protein in *Zc3h12c*^−/−^ BMDMs at early phase (0–4 h) but not at late phase (6–24 h) after activation (Fig. 2a, b, Supplementary Fig. 3a, b). In contrast, IL-6 production was unaffected at all timepoints by MCPIP3-deficiency, though it was little secreted at early phase (Fig. 2c, Supplementary Fig. 3c). Using TNF intracellular staining, we have observed similar TNF phenotype when comparing *Zc3h12c*^+/+^/*Zc3h12c*^−/−^ or *Zc3h12c*^fl/fl^/*Zc3h12c*^MΦΔ^ macrophages at early phase (Fig. 2d, e). In addition to TLR7 agonists, this early TNF secretion defect was also seen in MCPIP3-deficient macrophages activated by various TLR1/2/3/4 ligands (Fig. 2d, e). Besides BMDMs, we also examined MCPIP3-deficient Per. MΦs, and found a similar defect in early TNF production with R848 and Pam3CSK4 treatment (Fig. 2f). Besides TNF, IL-12 is a key inflammatory cytokine reported to be involved in early psoriasis pathogenesis^36^. We found that *Il12p40* transcripts were also decreased in R848-activated MCPIP3-deficient macrophages (Fig. 2g). Using intracellular staining, we observed ~50% reduction of IL-12p40 production by macrophages after R848, Pam3CSK4, or LPS activation (Fig. 2h, i). Although serum IFN-γ was reported to be affected by MCPIP3, we saw little IFN-γ production by either *Zc3h12c*^+/+^ or *Zc3h12c*^−/−^ BMDMs (Supplementary Fig. 3d)^24^. Thus, we conclude that MCPIP3 promotes TNF and IL-12p40 production in macrophages.

Macrophages balance diverse functions, such as their pro-inflammatory function and wound repair, by polarizing into M1 or M2 macrophages under different stimuli, such as LPS or IL-4^37^. To see if MCPIP3 affects M1/M2 polarization and associated functions, we subjected *Zc3h12c*^+/+^ and *Zc3h12c*^−/−^ BMDMs to LPS and IL-4 treatments in vitro, and measured several key macrophage polarization genes by qPCR. Judging by the signature genes such as *Arg1* and *Nos2*, the effects of R848 stimulation were more similar to those of LPS (Supplementary Fig. 3e). Similar to R848 treatment, *Zc3h12c* expression was upregulated in LPS-treated, but not in IL-4-treated BMDMs (Supplementary Fig. 3f). *ZC3H12C* expression was also higher in M1-conditioned human peripheral macrophages from PBMCs (Supplementary Fig. 3g). However, expression levels of *Arg1* and *Nos2* in remained unchanged in R848-treated MCPIP3-deficient BMDMs (Supplementary Fig. 3h). Instead, MCPIP3-deficient BMDMs expressed more *Ilr4a* transcripts and fewer *Ifnar1/Ifnar2* transcripts, which are signatures associated with alternatively activated macrophages^38^ (Supplementary Fig. 3h). Because TNF from M1-polarized macrophages is crucial for septic shock, we induced sepsis in *Zc3h12c*^+/+^/*Zc3h12c*^−/−^ and *Zc3h12c*^fl/fl^/*Zc3h12c* ^MΦΔ^ mice with LPS. We found that all mice had the same survival rate (Supplementary Fig. 3i). We also infected *Zc3h12c*^+/+^/*Zc3h12c*^−/−^ mice with *Listeria monocytogenes*, and found no differences in liver or spleen bacterial load (Supplementary Fig. 3j). Since M2 macrophages participate in tissue repair, we inflicted skin wounds on *Zc3h12c*^+/+^/*Zc3h12c*^−/−^ mice and found no difference in wound repair between them (Supplementary Fig. 3k). Thus, MCPIP3 is dispensable for classic M1/M2 macrophage functions.

### IL-6 production is enhanced in MCPIP3-deficient pDCs

**Fig. 3:**
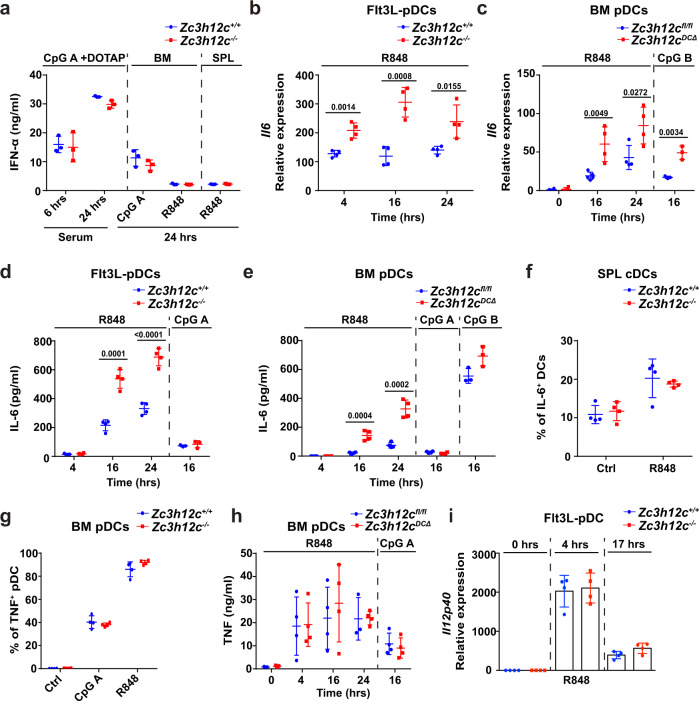
IL-6 production is enhanced in MCPIP3-deficient pDCs. **a** IFN-α secretion by *Zc3h12c*^+/+^(blue)/*Zc3h12c*^−/−^ (red) plasmacytoid dendritic cells (pDCs) was measured by ELISA after stimulation (*n* = 3 mice per group). **b** *Il6* expression in *Zc3h12c*^+/+^/*Zc3h12c*^−/−^ FL3TL-pDCs was measured by qPCR (*n* = 4 mice per group). **c** *Il6* expression in sorted *Zc3h12c* ^fl/fl^/*Zc3h12c*^DCΔ^ bone marrow (BM) pDCs was measured by qPCR (*n* = 4 mice per group). **d** IL-6 secretion by *Zc3h12c*^+/+^/*Zc3h12c*^−/−^ FL3TL-pDCs was measured by ELISA (*n* = 4 mice per group). **e** IL-6 secretion by sorted *Zc3h12c*^fl/fl^/*Zc3h12c*^DCΔ^ BM pDCs was measured by ELISA (*n* = 4 mice per group). **f** IL-6 secretion by *Zc3h12c*^fl/fl^/*Zc3h12c*^DCΔ^ splenic cDCs were measured by intracellular staining after R848 activation (*n* = 4 mice per group). **g** TNF production by *Zc3h12c*^+/+^/*Zc3h12c*^−/−^ BM pDCs was measured by intracellular staining after 4 h of stimulation (*n* = 4 mice per group). **h** TNF secretion by sorted *Zc3h12c*^fl/fl^/ *Zc3h12c*^DCΔ^ BM pDCs was measured ELISA (*n* = 4 mice per group). **i** *Il12p40* expression in *Zc3h12c*^+/+^/*Zc3h12c*^−/−^ FL3TL-pDCs was measured by qPCR (*n* = 4 mice per group). Data are representative of six (**a**), three (**b**–**e**), or two (**f**–**i**) independent experiments (mean ± S.D.; two-tailed Student’s *t* test). Source data are provided as a Source Data file.

Next, we examined the role of MCPIP3 in activated pDCs. The most well-known ability of pDCs is its massive production of IFN-α, especially under TLR9 agonist CpG A stimulation^39^. To determine if MCPIP3 regulates IFN-α production by pDCs, we stimulated *Zc3h12c*^+/+^*/Zc3h12c*^−/−^ or *Zc3h12c*^fl/fl^/*Zc3h12c*^DCΔ^ pDCs in vitro or in vitro with CpG A, CpG B, or R848, and measured their IFN-α production by intracellular staining and ELISA. We found that MCPIP3-deficient mice had normal serum IFN-α production after injection with CpG A associated with DOTAP in vivo (Fig. 3a). Further in vitro experiments showed that MCPIP3-deficiency did not affect IFN-α production from pDCs from bone marrow, spleen, or bone marrow-derived pDCs cultured with FL3TL (FL3TL-pDCs) (Fig. 3a, Supplementary Fig. 4e, f). MCPIP3 was also dispensable for immunity against acute and chronic LCMV viral infections^40^ (Supplementary Fig. 4g). Here we show that MCPIP3 is dispensable for IFN-α production by pDCs.

Besides IFN-I, pDCs were also known to produce cytokines such as IL-6 and TNF^39^. Since *Il6* was a well-known degradative target of the CCCH zinc finger family, we measured IL-6 expression in activated pDCs. We detected a consistent increase in *Il6* mRNA at all timepoints in *Zc3h12c*^−/−^ FL3TL-pDCs, with a ~3-fold increase at 16 h (Fig. 3b). We also found similar upregulation of IL-6 mRNA when comparing *Zc3h12c*^fl/fl^/*Zc3h12c*^DCΔ^ FACS-sorted bone marrow pDCs (Fig. 3c). Using ELISA, we found that IL-6 secretion was increased by ~3-fold in MCPIP3-deficient FL3TL-pDCs and sorted bone marrow pDCs after R848 stimulation (Fig. 3d, e). Interestingly, we found very little upregulation of *Zc3h12c*, *Il6* mRNA or IL-6 protein, with CpG A stimulation (Figs. 1g,  3d, e). Similarly, *Il6* mRNA in CpG B-treated MCPIP3-deficient pDCs was upregulated, along with a slight increase in IL-6 secretion (Fig. 3c, e). Although CD11c^+^ MHCII^+^ cDCs slightly upregulated *Zc3h12c* expression upon R848 activation, MCPIP3 was dispensable for their IL-6 or TNF production (Fig. 3f, Supplementary Fig. 4h–k). Despite MCPIP3’s regulation of TNF in macrophages, we saw no difference in TNF production by activated pDCs at early or late timepoints (Fig. 3g, h). IL-12p40 expression was also unaltered in MCPIP3-deficient pDCs (Fig. 3i). Here we demonstrate that MCPIP3 intrinsically represses IL-6 production in pDCs.

### Enhanced intradermal IL-6 alleviates psoriasiform skin inflammation

**Fig. 4:**
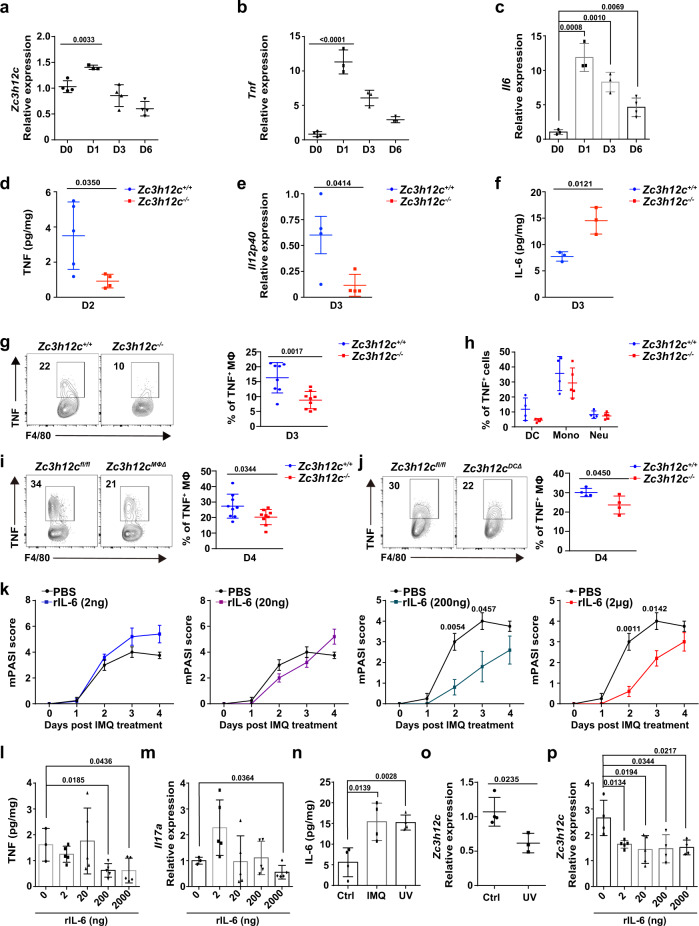
Enhanced intradermal IL-6 alleviates psoriasiform skin inflammation. **a–c** *Zc3h12c*, *Tnf*, and *Il6* expression in imiquimod (IMQ)-treated skin of WT mice at indicated days was measured by qPCR (**a**: *n* = 4 mice for day 0 and 3, *n* = 3 mice for day 1 and 6; **b**: *n* = 4 mice for day 0, *n* = 3 mice for day 1, 3, and 6; **c**: *n* = 3 mice for day 0, 1, and 3, *n* = 4 mice for day 6). **d** TNF secretion by IMQ-treated skin of *Zc3h12c*^+/+^(blue)/*Zc3h12c*^−/−^ (red)mice at day 2 was measured by ELISA after normalizing with weight (*n* = 5 mice per group). **e** *Il12p40* expression of IMQ-treated skin of *Zc3h12c*^+/+^/*Zc3h12c*^−/−^ mice at day 3 was measured by qPCR (*n* = 4 mice per group). **f** IL-6 secretion by IMQ-treated skin of *Zc3h12c*^+/+^/*Zc3h12c*^−/−^ mice at day 3 was measured by ELISA after normalizing with weight (*n* = 3 mice per group). **g**, **h** TNF production by infiltrated myeloid cells from *Zc3h12c*^+/+^/*Zc3h12c*^-−/−^ IMQ-treated skin on day 3 was measured by intracellular staining after R848 activation (**g**: *n* = 8 mice for *Zc3h12c*^+/+^; *n* = 9 mice for *Zc3h12c*^−/−^; **h**: *n* = 5 mice per group). **i** TNF production by infiltrated macrophages (CD45^+^ CD11b^+^ F4/80^+^ CD64^+^) from *Zc3h12c*^fl/fl^/*Zc3h12c*^MΦΔ^ IMQ-treated skin on day 4 was measured by intracellular staining after R848 activation (*n* = 9 mice per group). **j** TNF production by infiltrated macrophages (CD45^+^ CD11c^−^ CD11b^+^ F4/80^+^ CD64^+^) from *Zc3h12c*^fl/fl^/ *Zc3h12c*^DCΔ^ IMQ-treated skin on day 4 was measured by intracellular staining after R848 activation (*n* = 4 mice per group). **k** Psoriasis severity of IMQ-treated WT mice with rIL-6 injections were scored by mPASI daily (*n* = 5 mice per group). **l** TNF secretion by IMQ + rIL-6-treated skin at day 3 was measured by ELISA after normalizing with weight (*n* = 5 mice per group). **m** *Il17a* expression in IMQ + rIL-6 treated skin of WT mice at indicated days was measured by qPCR (*n* = 5 mice per group). **n** IL-6 secretion by treated skin was measured by ELISA after normalizing with weight (*n* = 4 mice per group). **o** *Zc3h12c* expression in UV-treated skin at 24 h was measured by qPCR (*n* = 5 mice per group). **p** *Zc3h12c* expression in IMQ + rIL-6-treated skin at day 4 was measured by qPCR (*n* = 5 mice per group). Data are representative of at least three independent experiments (mean ± S.D.; two-tailed Student’s *t* test). Source data are provided as a Source Data file.

Since inflammatory cytokines secreted by MCPIP3-deficient pDCs or macrophages were perturbed in vitro, we wish to validate these results in a relevant disease model in vivo. pDCs and macrophages were thought to participate in the early initiation of the psoriasis inflammatory loop^41–43^. However, the definitive role of pDCs in psoriasis is still under debate^44–47^. We found ~50% enrichment *Zc3h12c* transcripts in TLR7 agonist imiquimod (IMQ)-induced psoriasiform lesions of C57BL/6J (WT) mice (Fig. 4a)^48^. This increase in *Zc3h12c* mRNA at day 1 was correspondent to a massive increase in *Tnf* mRNA expression, a hallmark of early inflammatory loop in psoriasis (Fig. 4b). Similarly, we noticed an upregulation of *Il6* mRNA in IMQ-treated lesions (Fig. 4c).

Next, we measured secretion of inflammatory cytokines by psoriatic lesions of *Zc3h12c*^+/+^*/Zc3h12c*^−/−^ mice. Consistent with previous in vitro results, we found that *Zc3h12c*^−/−^ skin tissue released less TNF (Fig. 4d). IL-12p40 transcripts were also reduced (Fig. 4e). To identify the source of this TNF defect, we measured TNF secretion by skin-resident myeloid cells via intracellular staining. We found that TNF production by skin-infiltrated macrophages (CD11b^+^ F4/80^+^ CD64^+^) was decreased by ~50% in *Zc3h12c*^−/−^ mice, while TNF production in other myeloid cells remained unchanged (Fig. 4g, h). Consistent with the germline knockout mice, we observed a reduction of TNF secretion by skin-infiltrating *Zc3h12c*^MΦΔ^ macrophages (Fig. 4i). Here we show that MCPIP3-deficient skin-resident macrophages produce less TNF in psoriatic lesions.

We then investigated the physiological significance of pDC-derived IL-6 in the IMQ model. First, we examined pDC percentages from IMQ-induced psoriatic lesions at day 1, and found they were comparable between *Zc3h12c*^+/+^/*Zc3h12c*^−/−^ mice (Supplementary Fig. 4a, b). Consistently with previously published reports, pDC recruitment to skin peaked within 24 h after IMQ treatment (Supplementary Fig. 4c)^49^. pDCs and noncanonical DCs (nc-DCs)/transitional DCs (tDCs)/Axl^+^Siglec-6^+^ DCs (ASDCs) share similar surface markers, and these nc-DCs, tDCs, or ASDCs may have been confused for pDCs in previous psoriasis studies^50,51^. Using CX3CR1, a surface marker that differentiates murine pDCs and t-DCs, we confirmed that the CD11c^int^ B220^+^ Bst2^+^ SiglecH^+^ cells we identified in IMQ-induced lesions at day 1 were CX3CR1 negative (Supplementary Fig. 4d)^52^. Here, we confirm that bona fide pDCs are present early in IMQ-induced psoriatic lesions.

Similar to our pDC experiments in vitro, we found that *Zc3h12c*^−/−^ psoriatic skin released more IL-6 than that of *Zc3h12c*^+/+^ mice (Fig. 4f). To determine the effect of enhanced IL-6 by pDCs in vivo, we induced psoriatic lesions with IMQ on *Zc3h12c*^fl/fl^/*Zc3h12c*^DCΔ^ mice. Since some macrophages express CD11c, we gated on CD11c negative skin-infiltrating macrophages, and still found a ~25% reduction in TNF production (Fig. 4j). Here we show that MCPIP3-deficiency in dendritic cells could extrinsically suppress macrophage TNF.

To imitate the excessive IL-6 production by pDCs in MCPIP-deficient inflamed skin, we injected WT mice skin with recombinant IL-6 at the beginning of IMQ-induced psoriasis model. We found that intradermal rIL-6 injections above 200 ng significantly reduced the mPASI score of IMQ-treated WT mice (Fig. 4k). High doses of rIL-6 suppressed TNF secretion and *Il17a* transcripts in MCPIP3-deficient inflamed skin (Fig. 4l, m). Narrowband ultraviolet B (NB-UVB), a standard phototherapy to treat psoriasis, is known to induce copious amount of IL-6 in skin^53^. The amount of IL-6 induced by inflamed skin after UV irradiation is similar to that of IMQ model at day 1 (Fig. 4n). We found that *Zc3h12c* transcripts in WT mice skin were downregulated after UV irradiation (Fig. 4o). Similarly, rIL-6 injections could lower *Zc3h12c* transcripts in IMQ-treated skin (Fig. 4p). Here, we show that intradermal rIL6 injections protect mice against IMQ-induced psoriatic lesions.

### MCPIP3-deficiency protects mice from psoriatic skin inflammation

**Fig. 5:**
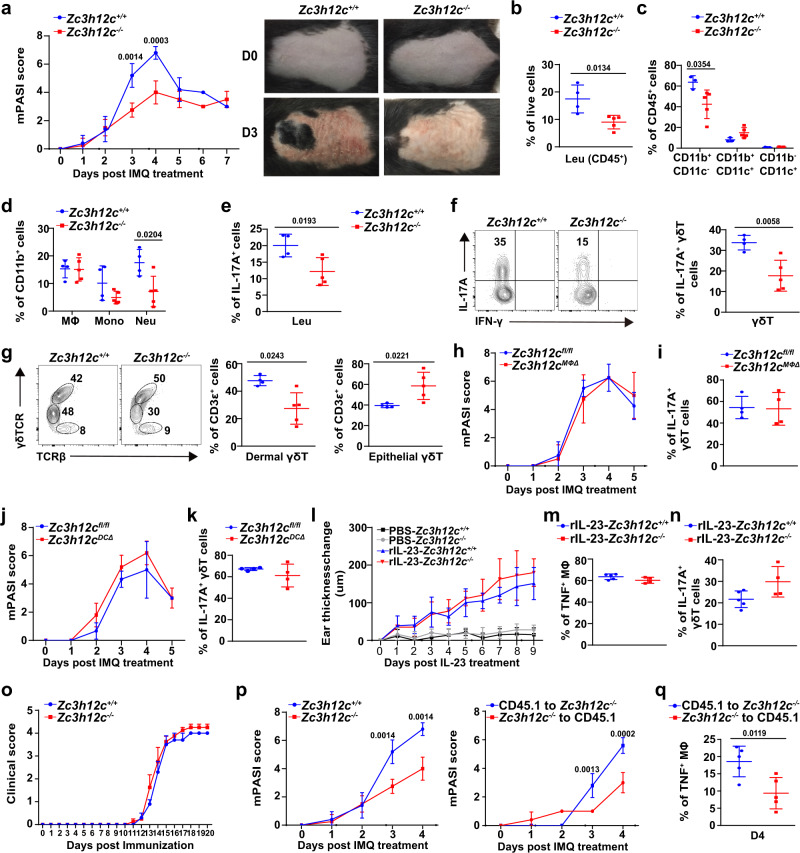
MCPIP3-deficiency protects mice from psoriatic skin inflammation. **a** Psoriasis severity of imiquimod (IMQ)-treated skin from *Zc3h12c*^+/+^(blue)/*Zc3h12c*^−/−^ (red)mice was measured daily by mPASI (*n* = 4 mice per group). Representative pictures of the treated back-skin were shown at right. **b–d** Percentages of infiltrated immune cells from IMQ-treated *Zc3h12c*^+/+^/*Zc3h12c*^−/−^ back-skin on day 3 were analyzed by flow cytometry (*n* = 4 mice for *Zc3h12c*^+/+^; *n* = 5 mice for *Zc3h12c*^−/−^). Cells were live gated as following: CD45^+^ leukocytes (Leu), CD45^+^ CD11b^+^ F4/80^+^ CD64^+^ macrophages (MΦ), CD45^+^ CD11b^+^ Ly6G^+^ neutrophils (Neu), CD45^+^ CD11b^+^ Ly6C^+^ monocytes (Mono). **e**, **f** IL-17A production by CD45^+^ leukocytes (**e**) and CD3ε^+^ γδTCR^+^ T cells (**f**) at *Zc3h12c*^+/+^/*Zc3h12c*^−/−^ IMQ-treated skin on day 3 was measured by intracellular staining (*n* = 4 mice for *Zc3h12c*^+/+^; *n* = 5 mice for *Zc3h12c*^−/−^). **g** γδ T-cell subset composition at IMQ-treated *Zc3h12c*^+/+^/*Zc3h12c*^−/−^ back-skin on day 3 was analyzed by flow cytometry (*n* = 4 mice for *Zc3h12c*^+/+^; *n* = 5 mice for *Zc3h12c*^−/−^). Dermal (γδTCR^+^ TCRβ^−^) and epithelial (γδTCR^+^ TCRβ^+^) γδ T-cell subsets were gated from live^+^ CD45^+^ CD3ε^+^. **h–k** Psoriasis severity of IMQ-treated *Zc3h12c*^fl/fl^/*Zc3h12c*^MΦΔ^/*Zc3h12c*^DCΔ^ mice were scored by mPASI daily (**h**, **j**), and IL-17A production by γδ T cells (**i**, **k**) at day 4 was measured (*n* = 4 mice per group). **l** Skin thickness of rIL-23-treated ears of *Zc3h12c*^+/+^/*Zc3h12c*^−/−^ mice was measured daily (*n* = 5 mice for *Zc3h12c*^+/+^; *n* = 4 mice for *Zc3h12c*^−/−^). **m** TNF production by infiltrated macrophages rIL-23-treated ears of *Zc3h12c*^+/+^/*Zc3h12c*^−/−^ mice at day 9 (*n* = 5 mice for *Zc3h12c*^+/+^; *n* = 4 mice for *Zc3h12c*^−/−^). **n** IL-17A production by γδ T cells at rIL-23-treated ear on day 9 was measured by intracellular staining (*n* = 5 mice for *Zc3h12c*^+/+^; *n* = 4 mice for *Zc3h12c*^−/−^). **o** EAE score of *Zc3h12c*^+/+^/*Zc3h12c*^−/−^ mice (*n* = 5 mice per group). **p** Psoriasis severity of the chimeras were scored by mPASI daily (*n* = 5 mice per group). **q** TNF production by infiltrated macrophages from the chimeras at day 4 was measured by intracellular staining after R848 activation (*n* = 5 mice per group). Data are representative of five (**a**–**g**), or two (**h**–**q**) independent experiments (mean ± S.D.; two-tailed Student’s *t* test). Source data are provided as a Source Data file.

Consistent with the perturbed TNF/IL-12/IL-6 secretion, we found that *Zc3h12c*^−/−^ mice had ~2-fold lower murine Psoriasis Severity Index (mPASI) score than *Zc3h12c*^+/+^ mice at days 3–4, the peak of IMQ-induced psoriatic lesions (Fig. 5a). We observed that the skin of *Zc3h12c*^−/−^ mice possessed significantly fewer white-scaly desquamations, which is the hallmark of psoriatic lesions (Fig. 5a). After isolating immune cells from the skin lesions, we observed that there were ~50% fewer infiltrated CD45^+^ leukocytes, CD11b^+^ CD11c^−^ myeloid cells, and Ly6G^+^ Ly6C^−^ neutrophils in *Zc3h12c*^−/−^ mice, when compared to *Zc3h12c*^+/+^ littermate controls (Fig. 5b–d). After initial activation by myeloid cells, subsequent IL-17A production by T cells further accelerates psoriasis pathogenesis^27^. We measured IL-17A production by skin-infiltrating CD45^+^ leukocytes and found ~50% decreased IL-17A production in *Zc3h12c*^−/−^ mice compared to *Zc3h12c*^+/+^ mice (Fig. 5e). We also examined γδ T cells, the predominant producers of IL-17A during psoriasis. IL-17A production by γδ T cells was decreased by ~1-fold in *Zc3h12c*^−/−^ mice compared to *Zc3h12c*^+/+^ mice (Fig. 5f). The percentage of dermal γδ T cells was also decreased by ~1-fold in *Zc3h12c*^−/−^ mice compared to *Zc3h12c*^+/+^ mice (Fig. 5g). Here we find that MCPIP3 promotes myeloid recruitment and T-cell activation in IMQ-induced psoriatic lesions.

However, we found no difference in mPASI clinical score, IL-17A production by γδ T cells or infiltrated immune cell numbers between *Zc3h12c*^fl/fl^/*Zc3h12c*^MΦΔ^/*Zc3h12c*^DCΔ^ mice (Fig. 5h–k, Supplementary Fig. 5a–e). Since *Zc3h12c*^MΦΔ^ and *Zc3h12c*^DCΔ^ mice could not exactly phenocopy *Zc3h12c*^−/−^ mice in the IMQ model, there is a possibility that unanticipated expression in cells other than pDCs or macrophages may contribute to this phenotype. CD11c^+^ MHCII^+^ CD11b^+^ dendritic cells (an heterogenous population consisting of cDC2 and MoDC) play an important role in psoriasis pathogenesis^54^. We confirm that few MoDCs were present at inflamed skin in the early days of IMQ model, and macrophages were ~75% monocyte-derived (Supplementary Fig. 3l).

Second, to exclude the possibility that MCPIP3 regulates IL-17A response in a T-cell-intrinsic manner, we used an recombinant IL-23-induced psoriasis model, which sidesteps initial activation by myeloid cell and activates T cells directly via IL-23^55^. We found the genetic ablation of *Zc3h12c* did not affect the inflamed ear skin thickness or other immune cell functions, including TNF and IL-17A secretion (Fig. 5l–n, Supplementary Fig. 5g–j). *Zc3h12c* mRNA was little expressed in various T-cell subsets (Fig. 1f, Supplementary Fig. 5k). Using a Th17-driven experimental autoimmune encephalomyelitis (EAE) model, we found that the EAE clinical score and Th17 responses in *Zc3h12c*^+/+^ and *Zc3h12c*^−/−^ mice were similar (Fig. 5o, Supplementary Fig. 5l). Thus, MCPIP3 regulates IL-17A production in a T-cell-extrinsic manner.

Lastly, to exclude the possibility that any expression of *Zc3h12c* in nonhematopoietic cells, such as keratinocytes, langerin cells, and tissue-resident macrophages, may play a role in psoriasis, we generated reverse chimeras from *Zc3h12c*^−/−^ mice (Supplementary Fig. 5m, n). We found that irradiated *Zc3h12c*^+/+^ mice reconstituted with *Zc3h12c*^−/−^ bone marrow (*Zc3h12c*^−/−^ to CD45.1) had similar mPASI score as *Zc3h12c*^−/−^ mice, whereas irradiated *Zc3h12c*^−/−^ mice reconstituted with *Zc3h12c*^+/+^ bone marrow (CD45.1 to *Zc3h12c*^−/−^) had similar mPASI score as *Zc3h12c*^+/+^ mice (Fig. 5p). Straight chimeras (*Zc3h12c*^−/−^ to CD45.1) phenocopied *Zc3h12c*^−/−^ mice in terms of infiltrated leukocytes; whereas reverse chimeras (CD45.1 to *Zc3h12c*^−/−^) did not (Supplementary Fig. 5o). Most importantly, skin-infiltrated macrophages from straight chimeras (*Zc3h12c*^−/−^ to CD45.1) exhibited ~50% decrease in TNF production, when compared with reverse chimeras (CD45.1 to *Zc3h12c*^−/−^) (Fig. 5q). Thus, MCPIP3-deficiency in cells of hematopoietic origin is necessary and sufficient to protect mice from psoriatic lesions. Collectively, these data suggest that simultaneous TNF repression and IL-6 enhancement, by MCPIP3-deficient macrophages and dendritic cells respectively, could protect mice against IMQ-induced psoriatic lesions.

### Exposure to pDC-derived IL-6 suppresses macrophage TNF and IL-12p40

**Fig. 6:**
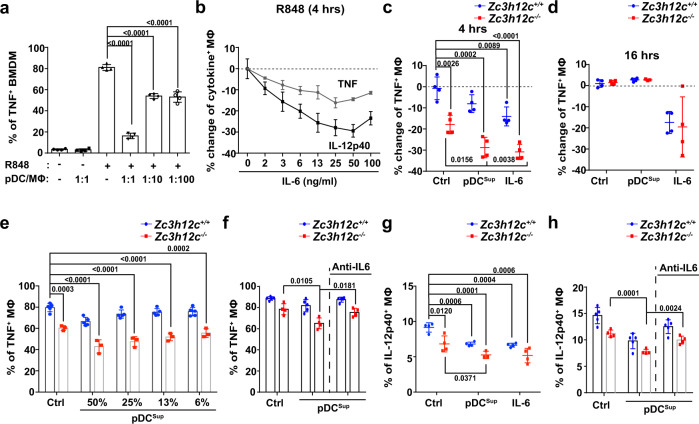
Exposure to pDC-derived IL-6 suppresses macrophage TNF and IL-12p40. **a** Sorted WT bone marrow-derived macrophages (BMDMs) and FL3TL-pDCs were placed into the same wells for 16 h before addition of R848. TNF production in macrophages was measured by intracellular staining in macrophages at 4 h (*n* = 4 mice per group). **b** WT BMDMs were treated with recombinant IL-6 at various concentrations, and macrophage TNF or IL-12p40 production was measured by intracellular staining at 4 h. Percentage changes was normalized against WT samples without IL-6 (*n* = 3 mice per group). **c**, **d** Sorted WT FL3TL-pDCs were treated with R848 for 24 h, and their supernatants (pDC^sup^) or rIL-6 (50 ng/ml) were added to *Zc3h12c*^+/+^(blue)/*Zc3h12c*^−/−^ (red)BMDMs along with R848. TNF production in macrophages at 4 h (**c**) or 16 h (**d**) was measured by intracellular staining. Percentage changes was normalized against *Zc3h12c*^+/+^ samples without pDC^sup^ or rIL-6 (*n* = 4 mice per group). **e** TNF production by *Zc3h12c*^+/+^/*Zc3h12c*^−/−^ BMDMs with sorted WT BM pDC supernatants was measured by intracellular staining after R848-treatment at 4 h (*n* = 5 mice per group). **f** TNF production by *Zc3h12c*^+/+^/*Zc3h12c*^−/−^ BMDMs with titrated concentrations of R848-treated sorted *Zc3h12c*^−/−^ FL3TL-pDC supernatants, with or without IL-6 neutralizing antibodies was measured by intracellular staining at 4 h (*n* = 3 mice for *Zc3h12c*^+/+^; *n* = 4 mice for *Zc3h12c*^−/−^). **g** IL-12p40 production by *Zc3h12c*^+/+^/*Zc3h12c*^−/−^ BMDMs with pDC^sup^ or rIL-6 was measured by intracellular staining at 4 h (*n* = 5 mice for *Zc3h12c*^+/+^; *n* = 4 mice for *Zc3h12c*^−/−^). **h** IL-12p40 production by *Zc3h12c*^+/+^/*Zc3h12c*^−/−^ BMDMs with titrated concentrations of R848-treated sorted *Zc3h12c*^−/−^ FL3TL-pDC supernatants, along with or without IL-6 neutralizing antibodies was measured by intracellular staining at 4 h (*n* = 4 mice per group). Data are representative of five (**c**, **d**), three (**g**), or two (**a**, **b**, **e**, **f**, **h**) independent experiments (mean ± S.D.; two-tailed Student’s *t* test). Source data are provided as a Source Data file.

It was previously shown that IL-6 from conventional dendritic cells could inhibit the production of inflammatory cytokines by inhibiting NFκb and inducing soluble p55 receptor for TNF^56^. Since macrophage TNF was downregulated in *Zc3h12c*^DCΔ^ psoriatic lesions (Fig. 4j), we hypothesize that pDCs could directly regulate macrophage TNF secretion. After co-culturing WT FL3TL-pDCs with WT BMDMs, we found that TNF secretion by R848-stimulated macrophages was blocked by ~5-fold with equal numbers of co-cultured pDCs (Fig. 6a). It was worth noting that TNF was decreased by ~30% with 1–10% pDCs, which was within range of pDC percentage amongst immune cells at psoriatic skin on day 1 (Fig. 6a, Supplementary Fig. 4a). To determine if IL-6 could suppress macrophage TNF, we treated WT BMDMs with various concentration of recombinant IL-6 along with R848, and then measured their TNF or IL-12p40 production by intracellular staining. We found that initial exposure to IL-6 could lower macrophage TNF by ~15%, and IL-12p40 production by ~30% (Fig. 6b). Next, we added supernatants of activated WT FL3TL-pDCs (pDC^sup^) to *Zc3h12c*^+/+^/*Zc3h12c*^−/−^ BMDMs, and then measured macrophage TNF production at 4 h by intracellular staining. As a comparison, we added recombinant IL-6. Supernatants from FL3TL-pDCs lowered TNF production of *Zc3h12c*^+/+^ and *Zc3h12c*^−/−^ macrophages by ~15%, similar to IL-6 (Fig. 6c). This suppressive effect was absent at late-phase of macrophage activation (Fig. 6d). We repeated the same experiments with FACS-sorted BM pDCs, and found that the difference between *Zc3h12c*^+/+^/*Zc3h12c*^−/−^ TNF^+^ macrophages now reached ~50% (Fig. 6e). We were finally able to recreate this substantial TNF reduction in vitro, similar to what was observed in vivo at psoriatic lesions (Fig. 4g). Similar to WT pDCs, *Zc3h12c*^−/−^ pDC supernatant suppressed macrophage TNF, and this suppressive effect was abolished with addition of neutralizing anti-IL-6 monoclonal IgG antibodies (Fig. 6f). pDC supernatants suppressed macrophage IL-12p40 in a similar manner (Fig. 6g, h). Here we show in vitro that early exposure to pDC-derived IL-6 suppresses macrophage TNF and IL-12p40 production.

pDC secret copious amount of IFN-I, which was known to regulate TNF production in various cell types^3^. IFN-α suppression of macrophage TNF was reported under FcRε ligation and Pam3CSK stimulation, but the effect under R848 was not explored^57^. First, we observed that supernatant from CpG A-activated FL3TL-pDCs could not suppress macrophage TNF at all (Supplementary Fig. 4l). Second, we could not find any IFN-α suppression of TNF in R848-activated macrophages with ELISA or intracellular staining, even at high concentrations (1 mg/ml) (Supplementary Fig. 4m, n). Lastly, we induced psoriasis with IMQ on double transgenic *Ifnar1*^−/−^*: Zc3h12c*^−/−^ mice. We found that *Ifnar1*^−/−^: *Zc3h12c*^−/−^ mice maintained the same phenotype as *Ifnar1*^−+/+^: *Zc3h12c*^−/−^ mice, in terms of ear thickness, infiltrated immune cells and IL-17A production (Supplementary Fig. 4o, p). Our data show that suppression of R848-activated macrophage TNF by pDCs is independent of IFN-I signaling.

### MCPIP3 directly degrades IL-6 and Regnase-1 mRNAs

**Fig. 7:**
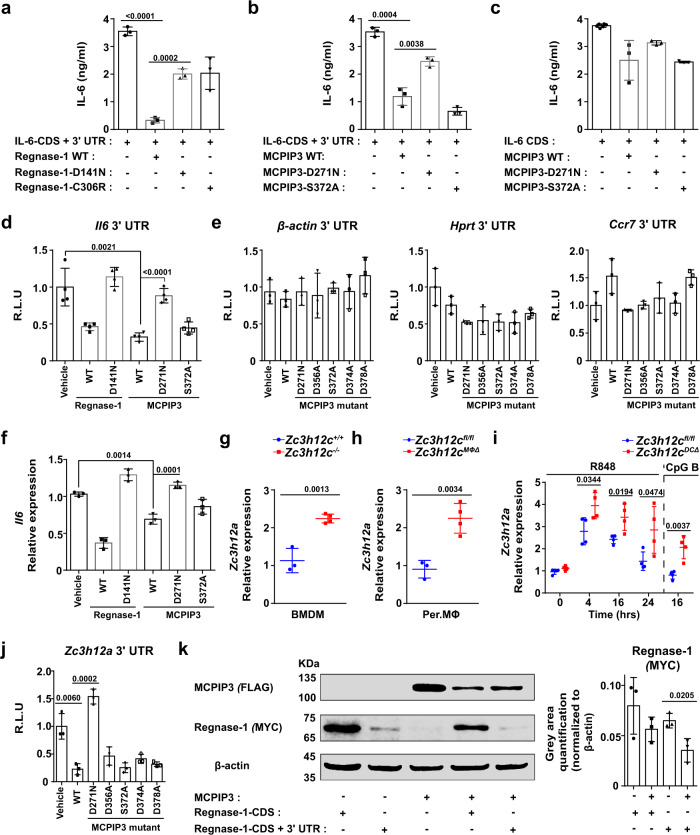
MCPIP3 directly degrades IL-6 and Regnase-1 mRNAs. **a–c** 293T cells were co-transfected with Regnase-1 (**a**) or MCPIP3 (**b**, **c**), and IL-6 CDS with 3′ UTR (**a**, **b**) or without 3′ UTR (**c**). After 48 h, IL-6 in supernatants were measured by ELISA (*n* = 4 replicates for IL-6 CDS only; *n* = 3 replicates for all other groups). **d** Degradation of *Il6* mRNA by MCPIP3 was measured by a luciferase reporter system. 3′ UTR of *Il6* were inserted downstream of the luciferase gene. These reporter plasmids were then transfected into 293T cells, along with empty, MCPIP3 or mutant MCPIP3 overexpression plasmids (*n* = 4 replicates). **e** 3′ UTR of negative control genes (*β-actin, Hprt, Ccr7*) were inserted downstream of the luciferase gene. **f** 293T cells were co-transfected with MCPIP3 and IL-6 CDS with 3′ UTR. After 48 h, *Il6* mRNA in transfected cells were measured by qPCR (*n* = 3 replicate wells). **g** *Zc3h12a* (the gene encoding Regnase-1) expression in sorted *Zc3h12c*^+/+^/*Zc3h12c*^−/−^ bone marrow-derived macrophages (BMDMs) was measured by qPCR (*n* = 3 mice for *Zc3h12c*^+/+^; *n* = 4 mice for *Zc3h12c*^−/−^). **h** *Zc3h12a* expression in purified *Zc3h12c*^fl/fl^/*Zc3h12c*^MΦΔ^ peritoneal macrophages (Per. MΦ) was measured by qPCR (*n* = 3 mice for *Zc3h12c*^+/+^; *n* = 4 mice for *Zc3h12c*^−/−^). **i** *Zc3h12a* expression in sorted *Zc3h12c*^fl/fl^/*Zc3h12c*^DCΔ^ bone marrow (BM) pDCs was measured by qPCR (*n* = 4 mice per group). **j** Degradation of *Zc3h12a* mRNA by MCPIP3 was measured by a luciferase reporter system (*n* = 3 replicates). **k** 293T cells were transfected with FLAG-tagged MCPIP3 together with MYC-tagged Regnase-1. After 48 h, whole cell lysates were immunoprecipitated with anti-MYC or FLAG antibodies. Gray area quantification of the Regnase-1-MYC was calculated by ImageJ, after normalizing with β-actin bands (*n* = 3 replicate bands). The samples were derived from the same experiment and that gels/blots were processed in parallel. Data are representative of at least three independent experiments (mean ± S.D.; two-tailed Student’s *t* test). Source data are provided as a Source Data file.

Since MCPIP3 contains putative zinc finger CCCH RNA-binding and NYN RNase domains, we sought to identify specific degradative targets of MCPIP3 in pDCs and macrophages. Because we already found that IL-6 protein and mRNA was upregulated in MCPIP3-deficient pDCs, we hypothesized that MCPIP3 might directly degrade IL-6 mRNAs. For this purpose, we mutated possible enzymatically active sites (D271N, D271A, D356A, S372A, D374A, and D378A) at the predicted RNA-binding groove of NYN nuclease domain of MCPIP3. As a positive control, we utilized Regnase-1, a known IL-6 mRNA degrader, and its functionally inactive D141N or C306R mutants (Fig. 7a).

First, we measured secreted IL-6 in the supernatant after co-transfecting 293T cells with IL-6 and MCPIP3 overexpression plasmids. MCPIP3 could lower IL-6 concentrations in the supernatant, albeit not as efficient as Regnase-1 (Fig. 7b). Overexpression by MCPIP3 D271N mutant was able to rescue IL-6 secretion, whereas S372A mutant could not (Fig. 7b). In a negative control using *Il6* CDS without 3′UTR, neither MCPIP3 nor its mutants could not have any effects on IL-6 concentrations (Fig. 7c). Second, we cloned the IL-6 3′UTR region into a luciferase reporter system, along with a MCPIP3 overexpression plasmid. Similar to Regnase-1, MCPIP3 was able to abolish reporter signals from IL-6 3′UTRs constructs (Fig. 7d). Consistently, MCPIP3 D271N mutant failed to abolish IL-6 reporter signals (Fig. 7d). To address the concern that MCPIP3 may be an unspecific RNase, we utilized 3′UTR of several negative control mRNAs such as *β-actin, Hprt*, or *Ccr7*. We found that their luciferase activity was unaffected by MCPIP3 overexpression (Fig. 7e). Lastly, total IL-6 mRNA was reduced in presence of MCPIP3 (Fig. 7f). These results suggest that MCPIP3 uses its NYN nuclease domain to degrade IL-6 mRNA by binding to its 3′ UTR.

Besides IL-6, we found that a close family member, *Zc3h12a* (the gene encoding Regnase-1/MCPIP1/ZC3H12A), was upregulated in *Zc3h12c*^−/−^ BMDMs (Fig. 7g). By comparison, the mRNA expression of MCPIP2 (*Zc3h12b)* and MCPIP4 (*Zc3h12d)* were not affected in MCPIP3-deficient macrophages (Supplementary Fig. 6b, c). To exclude the possibility that MCPIP1’s upregulation is due to genetic compensation from point mutations in *Zc3h12c*^−/−^ mice, we examined *Zc3h12a* mRNA in Per. MΦs of *Zc3h12c*^MΦΔ^ mice, in which an entire exon was deleted. *Zc3h12a* mRNA was also upregulated in *Zc3h12c*^MΦΔ^ macrophages (Fig. 7h). In pDCs, we detected an increase of *Zc3h12a* mRNA, albeit only after R848 or CpG B activation (Fig. 7i). Here we demonstrate that Regnase-1 mRNA is upregulated in MCPIP3-deficient macrophages and pDCs.

To determine whether MCPIP3 could bind and degrade Regnase-1 mRNA, we cloned the *Zc3h12a* 3′UTR region into a luciferase reporter system, along with a MCPIP3 overexpression plasmid. We found that 293T cells transfected with *Zc3h12a* 3′UTRs showed ~4-fold less luciferase activity in the presence of MCPIP3 (Fig. 7j). The MCPIP3 D271N mutant was able to restore *Zc3h12a* reporter signals, suggesting this could be a key site for nuclease activity (Fig. 7j). Second, we constructed tagged Regnase-1 (MYC) and MCPIP3 (FLAG), and examined Regnase-1 protein level in the presence of MCPIP3 protein. When MCPIP3 was co-transfected with *Zc3h12a* CDS with its 3′UTR, less amount of Regnase-1 protein was found (Fig. 7k). In contrast, presence of MCPIP3 did not affect Regnase-1 when co-transfecting with *Zc3h12a* CDS only without its 3′UTR (Fig. 7k). Our results suggest that MCPIP3 specifically binds to *Zc3h12a* 3′ UTR, and degrades Regnase-1 mRNA with its nuclease domain.

### MCPIP3 regulates cytokine secretion via Regnase-1 and NFκB pathway

**Fig. 8:**
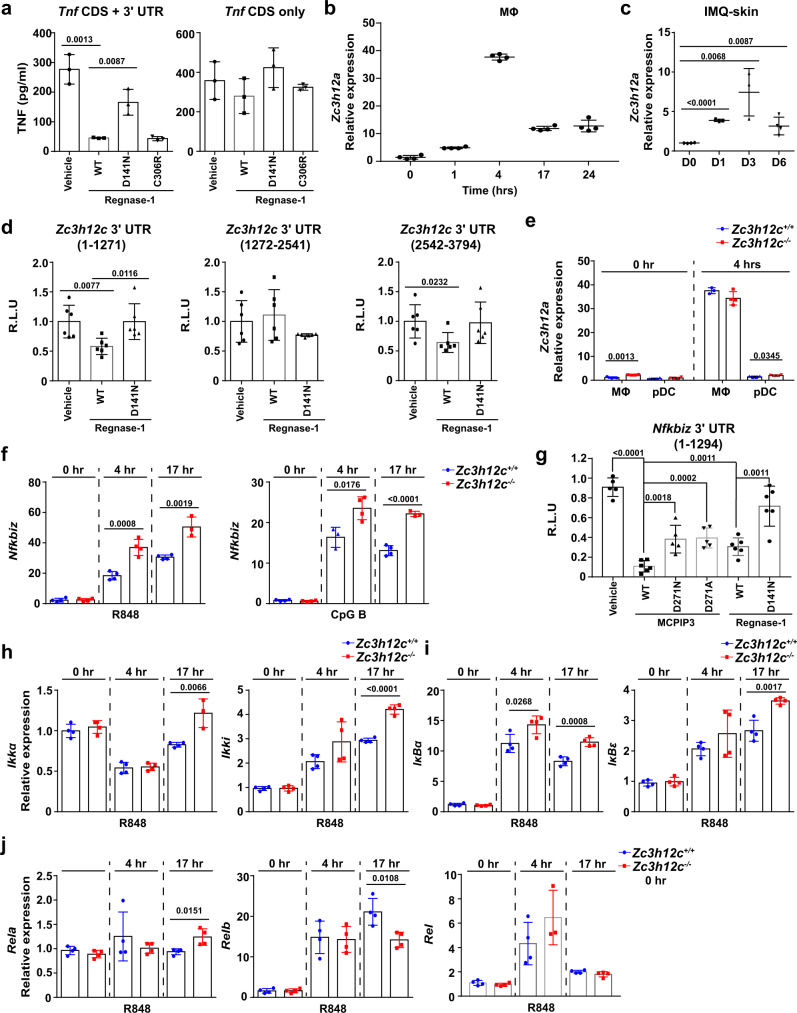
MCPIP3 regulates cytokine secretion via Regnase-1 and NFκB pathway. **a** 293T cells were co-transfected with Regnase-1, TNF-CDS with 3′ UTR or without 3′ UTR. After 48 h, TNF in supernatants were measured by ELISA (*n* = 3 replicate wells). **b** *Zc3h12a* expression in WT BMDMs was measured by qPCR (*n* = 5 mice per group). **c** *Zc3h12a* expression in IMQ-treated skin of WT mice at indicated days was measured by qPCR (*n* = 3 mice per group). **d** Degradation of *Zc3h12c* mRNA by Regnase-1 was measured by a luciferase reporter system. *Zc3h12c* 3′UTR (separated into three segments) were inserted downstream of the luciferase gene (*n* = 6 replicates). **e** *Zc3h12a* expression in *Zc3h12c*^+/+^/*Zc3h12c*^−/−^ FL3TL-pDCs and BMDMs was measured by qPCR (*n* = 4 mice per group). **f** *Nfkbiz* expression in *Zc3h12c*^+/+^/*Zc3h12c*^−/−^ FL3TL-pDCs was measured by qPCR (*n* = 4 mice per group). **g** Degradation of *Nfkbiz* mRNA by MCPIP3 was measured by a luciferase reporter system (*n* = 6 replicates). **h–j** Expression of indicated NFκB family members in *Zc3h12c*^+/+^/*Zc3h12c*^−/−^ FL3TL-pDCs was measured by qPCR (*n* = 4 mice per group). Data are representative of three (**a**–**e**) or two (**f**–**j**) independent experiments (mean ± S.D.; two-tailed Student’s *t* test). Source data are provided as a Source Data file.

Regnase-1 exerts its TNF suppression by degrading mRNA of NFκB signaling molecules such as c-Rel and IκBζ, and possibly TNF itself^8,14,20,58^. Regnase-1-deficient mice exhibited excessive TNF secretion during septic shock, and Regnase-1 could bind to a stem loop at CDE37 region of *Tnf* 3′UTR in luciferase reporter assays^14,58^. We confirmed the direct degradation of TNF by Regnase-1 by measuring secreted TNF with ELISA, after co-transfecting *Zc3h12a* and *Tnf* overexpression plasmids (Fig. 8a). Thus, MCPIP3 could promote TNF expression by degrading Regnase-1, a negative TNF regulator.

It remains puzzling how TNF and IL-6 was regulated distinctively by MCPIP3 in macrophages and pDCs. In macrophages, Regnase-1 upregulation coincided with the that of MCPIP3 after 4 h (Fig. 8b). In psoriatic skin, Regnase-1 upregulation peaked at day 3, rather than day 1 for MCPIP3 (Fig. 8c). To determine if Regnase-1 could degrade MCPIP3 mRNA, we utilized luciferase reporter plasmids inserted with MCPIP3 3′UTR, along with Regnase-1 plasmids. We found that Regnase-1 was able to decrease luciferase reporter activity by binding to *Zc3h12c* 3′ UTR (Fig. 8d). In comparison, Regnase-1 expression in naïve or activated pDCs was minimal (~15-fold less) when compared to activated macrophages (Fig. 8e). Here we demonstrate that Regnase-1 could degrade MCPIP3 in macrophages, but is little expressed in pDCs.

Besides cytokines and family members, we screened the mRNA expression levels of transcriptional regulators such as A20, HuR, TTP (ZFP36), IRF3/7, and NFκB signaling pathway molecules in *Zc3h12c*^−/−^ macrophages and pDCs (Supplementary Figs. 6, 7). We found little difference in macrophages, possibly because of the transient expression of MCPIP3 and compensation by Regnase-1 (Supplementary Fig. 6a). Thus, we focused on pDCs, because they expressed the highest level of MCPIP3, and little Regnase-1. We found an atypical NFκB famly member, IκBζ (encoded by *Nfkbiz*) was upregulated in MCPIP3-deficient pDCs at early and late timepoints of activation (Fig. 8f). To determine if *Nfkbiz* is a direct degradative target of MCPIP3, we utilized the same luciferase reporter system. MCPIP3 could significantly decrease luciferase signals linked to *Nfkbiz* 3’UTR, at an even better efficiency than Regnase-1 (Fig. 8g). In addition, expression of several members of the NFκB family were affected by MCPIP3-deficiency in pDCs (Fig. 8h–j, Supplementary Fig. 7b). However, most of these changes occurred at late phase, rather than early phase after activation (Fig. 8h–j). Besides R848, we also found similar results with CpG B activation. (Supplementary Fig. 7c). In contrast, MCPIP3 did not seem to regulate c-Rel expression, another known target of Regnase-1 (Fig. 8j). Here we conclude that MCPIP3 could regulate NFκB pathway by directly degrading IκBζ mRNA.

## Discussion

Herein, we found that MCPIP3 promotes psoriatic skin inflammation by differentially regulating TNF, IL-6 and IL-12p40 secretion by macrophages and pDCs. In macrophages, TNF regulation during early-phase and late-phase is quite distinct, and is differentially regulated by IκBα and A20^59^. Similarly, Regnase-1 and roquin 1 promote the decay of inflammatory mRNAs at different phases of the inflammatory response^14^. Regnase-1 controls the early phase of inflammation via the cleavage and degradation of translationally active mRNAs, whereas roquin 1 controls the late phase of inflammation by removing translationally inactive mRNAs^14^. Our observations are consistent with a previous report which found no difference in MCPIP3-deficient macrophage TNF secretion after 8 h (late-phase). Unfortunately, this report did not examine any early timepoints^24^. Collectively, our results suggest early-phase of macrophage activation could be a key to initiate autoimmune skin inflammation. However, a defect in macrophage TNF could not fully account for the phenotype in MCPIP3-deficient mice.

Although the pro-inflammatory roles of TNF and IL-12 are well-established, the immunosuppressive role of IL-6 was overlooked in autoimmunity. IL-6 could switch between inflammatory and anti-inflammatory roles, depending on specific scenarios^2^. Mechanistically, IL-6 signaling via the soluble IL-6R (trans-signaling) is pro-inflammatory, whereas classic signaling via the membrane-bound receptor is regenerative and anti-inflammatory^60^. In psoriasis, IL-6 has been long associated with disease pathogenesis^61^. IL-6 is produced by keratinocytes, fibroblasts, endothelial cells, dendritic cells, macrophages, and T-helper type 17 cells^27,61^. However, genetic deletion of IL-6 can worsen psoriasis in certain animal models^62^. Clinically, IL-6 targeted blockade are ineffective or even detrimental in psoriasis and other indications^6^. Anti-IL-6 receptor antibody tocilizumab has been reported to induce a psoriasis-like disease in some patients^63^. Thus, we hypothesize that IL-6 may play differential roles during early and late stages of psoriasis pathogenesis. IL-6 may be anti-inflammatory during early phase of psoriasis, when pDCs were heavily recruited. During late phase, IL-6 stimulated proliferation of cultured human keratinocytes, a major hallmark of psoriatic lesions^61^. Skin inflammation was also abrogated in IL-6-deficient mice with recombinant IL-23-induced psoriasiform lesions^64^. IL-6 may synergize with IL-17C and TNF to drive a psoriasis-like signature from keratinocytes^62^. Therefore, IL-6 could be pro-inflammatory during late phase of psoriasis pathogenesis.

While the aberrant production of IFN-α by pDCs clearly promotes IFN-I-driven autoimmunity such as systemic lupus erythematosus, the exact role of pDCs in psoriasis is still puzzling and controversial^50^. In addition, pDC-derived IFN-α was reported to be involved in pro-inflammatory processes and promote wound healing^65–67^. However, genetic ablation of pDCs or IFN-I receptors did not affect the severity of imiquimod-induced psoriatic plaque formation in mice^46,47^. It is puzzling that activated pDCs were recruited to inflamed skin, but played non-essential roles in psoriasis. Because IFN-I was long-seen as the exclusive functional cytokine secreted by pDCs, most studies focused only on IFN-I signaling. pDC-derived IL-6 may offer a plausible mechanism to explain pDC’s puzzling role in psoriasis.

The conclusion that MCPIP3 may promote skin inflammation is unusual. However, our characterization of MCPIP3 protein function per se is quite similar to its family members. First, TPP, Roquin and Regnase-1 are potent degraders of IL-6 mRNA^8^. We found that MCPIP3 could also degrade IL-6 mRNAs. Second, Regnase-1 is known to regulate NFκB pathway, and we found MCPIP3 could directly degrade IκBζ^14,20^. Third, Regnase-1 was previously proposed to be MCPIP3’s degradative target, and we found similar results^24^. Nevertheless, the physiological function of the RNase is dependent on its degradative targets. TNF and IL-12p40 are known degradative target of Regnase-1^15^. IκBζ-deficient mice showed severe skin irratation, and IκBζ is reported to promote IL-6 expression, while suppressing TNF^68–70^. Therefore, MCPIP3 may promote TNF/IL-12 via Regnase-1, and suppress IL-6 via direct mRNA degradation and indirectly via IκBζ.

Our study does not fully explain how MCPIP3 expression is dynamically regulated, or how does MCPIP3 achieve its degradative specificity. First, the spatiotemporal expression of MCPIP3/Regnase-1 in macrophages and pDCs seemed to be designed for maximum synergy to promote inflammation. The rapid degradation of MCPIP3 by Regnase-1 might explain why MCPIP is dispensable for late phase TNF secretion in macrophages. Similarly, the lack of Regnase-1 might explain how MCPIP3 is dispensable for TNF production in pDCs. Besides Regnase-1, a recent report found that macrophages upregulated MCPIP3 upon TLR3 agonist Poly:IC stimulation, and MCPIP3 expression was dependent on IRF3/7 but not NFκB^24^. We found robust MCPIP3 upregulation after activation by TLR4/7/9 ligands in macrophages and pDCs. Our results were consistent because TLR7/9 signaling signals were dependent on IRF3/7^39^. Second, additional binding partners may be required to increase MCPIP3 specificity to its degradative targets, as mRNA 3′ UTRs are not unique enough to allow the precise recognition of individual mRNAs. For example, Regnase-1 requires the helicase activity of UPF1, as well as roquin 1 for the efficient degradation of IL-6 mRNA^14^. We have consistently shown that MCPIP3 could bind and degrade IL-6, Regnase-1, or IκBζ mRNA by their 3′UTRs, but not other negative control genes. However, a previous report concludes that MCPIP3’s mRNA degradative activity is unspecific, as MCPIP3 degraded all their samples including negative controls^24^. This experimental discrepancy could be due to the presence of unknown co-factors that facilitate or specify MCPIP3’s recognition of mRNAs.

Overall, our findings demonstrate that RNase MCPIP3 orchestrates TNF and IL-6 secretion by macrophages and pDCs to exert a surprisingly pro-inflammatory role in psoriatic lesions. This suggests that MCPIP3 could be a potential inhibitory target to treat psoriasis and other autoimmune diseases.

## Methods

### Human samples

Blood sample collection from healthy donors: Peripheral blood samples were collected from healthy donors, after taking written informed consent, in accordance with the Declaration of Helsinki and as recommended and approved by the institutional review board of CSIR-Indian Institute of Chemical Biology, Kolkata, India.

Immune cell subset isolation: PBMC were isolated from whole blood by sucrose density-gradient centrifugation using Hisep LSM (Ficoll). The various immune cell subsets (B cells, CD4^+^ T cells, CD8^+^ T cells, monocytes, CD1c^+^ cDCs, pDCs) were isolated by magnetic immune-selection using CD19, CD4, CD8, CD14, CD1c, and BDCA4 microbeads (Miltenyi Biotec) respectively. For monocyte-derived DC (moDC) generation, CD14^+^ monocytes were cultured for 5 days at a density of 0.5 million cells per well, in the presence of GM-CSF (20 ng/ml) and IL-4 (10 ng/ml). For monocyte-derived macrophages (M0 macrophage) generation, CD14^+^ monocytes were seeded at a density of 0.25 million cells per well (48-well plate), cultured for 7 days in the presence of M-CSF (20 ng/ml). After 7 days, M0 macrophage were treated with IFN-γ (20 ng/ml) and LPS (100 ng/ml) to generate M1 macrophage; or with IL-4 (20 ng/ml) to generate M2 macrophages.

Gene expression assays: Total RNA was isolated from the immune cell subsets using TRIzol reagent (Invitrogen, USA) and cDNA prepared (MultiScribe Reverse Transcriptase cDNA kit, Invitrogen) following manufacturer’s protocol. Quantitative PCR was carried out (QuantStudio 3 PCR machine) for *Zc3h12c* gene, with each sample being run in triplicates and normalized against 18 S rRNA as the housekeeping gene.

### Transgenic mice

C57BL/6J (WT), B6.SJL-*Ptprc*^*a*^*Pepc*^*b*^/BoyJ (CD45.1^+^), B6.129S2-Ifnar1^tm1Agt/Mmjax^ *(Ifnar1*^−/−^), B6.129P2-Lyz2tm1(cre)^Ifo/J^ (*Lyz2-Cre*) and B6.Cg-Tg (*Itgax*-cre)^1-1Reiz/J^ (*Itgax-Cre*) were purchased from The Jackson Laboratory. *Zc3h12c*^−/−^ and *Zc3h12c*^fl/fl^ mice were generated in-house and could be provided upon reasonable request. *Zc3h12c*^fl/fl^ mice were generated with technical expertise from GemPharmatech Co. Ltd. Age and sex-matched mice between 6 and 10 weeks of age were used. All mice were bred and maintained in specific-pathogen free (SPF) at GemPharmatech Co. Ltd. and BSL3 facilities at SYSU. This study complied with all relevant ethical regulations for animal testing research according to the institutional guidelines and protocols was approved by the Animal Ethics Committee of Sun Yat-sen University.

Generation of *Zc3h12c*^−/−^ germline knockout mice: In short, Cas9 mRNA and CRISPR-Cas9 sgRNA were co-injected into zygotes. −1 bp indel frame shift mutation was obtained on exon 3, resulting in a premature stop codon on exon 4. Mice were genotyped by sequencing PCR products amplified with the following primers (*Zc3h12c*-Fwd: ACT TGA ATT TGG GTT TCA TTG TGC T, *Zc3h12c*-Rvs: TCC CTA GAG TGA CAA GGC CC). To avoid any off-target mutations, the resulting mice were backcrossed with C57BL/6J for at least five generations, and the top predicted ten CRISPR-Cas9 off-target sites were sequenced and verified to be unmutated. Whole exon sequencing was also performed to ensure the only *Zc3h12c* was targeted.

Generation of *Zc3h12c*^fl/fl^ conditional knockout mice: Cas9 mRNA and sgRNA were co-injected into zygotes. sgRNA directed Cas9 endonuclease cleavage at intron 2–3 and intron 3–4, and created a DSB (double-strand break). Such DSBs would be repaired, and result in loxP sites inserted into intron 2–3 and intron 3–4 respectively by homologous recombination. Mice were genotyped via PCR (*Zc3h12c*-fl-Fwd: ACC AGG TTA GTG CTA ACA GTG G, *Zc3h12c*-fl-Rvs: CAC TGC ATG GCC TAC CCT GA). The insertion of loxP sites were confirmed by expected PCR bands of 217 bp, as the wildtype band was 122 bp. Cell-specific deletion of *Zc3h12c* allele was obtained after Cre-mediated recombination after crossed with *Lyz2-Cre* or *Itgax-Cre*. Deletion in both Cre lines were confirmed PCR and qPCR with FACS-sorted macrophages or dendritic cells.

### Generation of chimeras

CD45.1^+^ or *Zc3h12c*^−/−^ mice were lethally irradiated with 950 Rad and then injected *i.v*. with 5 × 10^6^ bone marrow cells harvested from age and sex-matched CD45.1^+^ or *Zc3h12c*^−/−^ mice. The mice were fed with 0.5 g/L neomycin sulfate hydrate (BBI life sciences, Cat#A610366-0100) for 2 weeks and then rested for at least an additional 4 weeks to allow reconstitution of immune cells.

### Induction of psoriasiform lesions

IMQ: Mice between 8 and 12 weeks of age received a daily topical dose of 62.5 mg (3.125 mg of the active compound) of Imiquimod (IMQ) cream (5%; Aldara; 3M Pharmaceuticals) on the shaved back or ears for consecutive days. Aldara (IMQ) cream (Pharmaceutical grade) was obtained from iNova (3M Health Care Limited). Back-skin samples were excised using 5 mm biopsy punch and extracted for lymphocytes. Skin punches were also flash frozen, and stored at liquid nitrogen before isolation for total RNA. To score the severity of inflammation of the back skin, an objective scoring system was developed based on the clinical Psoriasis Area and Severity Index (PASI), except that for the mouse model the affected skin area is not taken into account in the overall score (mPASI). Erythema and scaling were scored independently on a scale from 0 to 4: 0, none; 1, slight; 2, moderate; 3, marked; or 4, very marked. The level of erythema was scored using a scoring table with red taints. The cumulative score (erythema plus scaling) served as a measure of the severity of inflammation (scale 0-8). All measurements were performed blinded, without knowledge of mice genotype.

Recombinant IL-23: mice ears were intradermally injected with 20 µl of vehicle (PBS) or 500 ng of recombinant mouse IL-23 (Cat# CT028-M08H,Sino Biological) using a 33-gauge needle. Injections were repeated on alternate days for a total of five to seven doses. Ear thickness was measured on days without injection with 0.01-mm-thickness gauge dial micrometer (TECLOCK JAPAN). All measurements were performed blinded, without knowledge of mice genotype. Mice were sacrificed and skin samples were collected using the same procedures as IMQ-treated mice.

Recombinant IL-6: mice back skins were intradermally injected with 100 µl vehicle (PBS) or 2ug/200 ng/20 ng/2 ng (in 100ul) of recombinant mouse IL-6 (Cat# 50136-MNAE, Sino Biological) for 4 days, starting from the day of IMQ application.

### Wound healing and septic shock

Skin healing: Full-thickness wounds were created by excision of 4-mm punches on shaven back skin. Prior to the surgery, aged-matched mice were anesthetized with isoflurane and subcutaneous injections of buprenorphine(0.5 mg/kg) were given as analgesics. Wound area was measured by tracing onto a glass slide at the indicated timepoints. Slides were scanned and area calculated with ImageJ software.

Septic shock: LPS from *E. coli* 055:B5 (Cat#L12880,Sigma-Aldrich) and D-(+)-Galactosamine hydrochloride(CAS# 1772-03-8, Macklin) were diluted in pyrogen-free saline. A combination of LPS (5 μg per kg body weight) and D-gal (400 mg per kg body weight) was injected intraperitoneally. The animal’s survival was scored for mortality between 0 and 24 h.

### EAE induction

For active EAE induction, age- and sex-matched mice were immunized s.c. with MOG_35-55_ (Genscript, purity >98%) peptide (300 μg) emulsified in CFA containing 4 mg/ml heat-killed MTB (Cat#7001,Chondrex). 200 ng Pertussis toxin (Cat#181239A1, List Biological Laboratories) dissolved in PBS was administered *i.p*. on days 0 and 2. Mice were examined daily and scored for disease severity using the standard scale: 0, no clinical signs; 1, limp tail; 2, paraparesis (weakness, incomplete paralysis of one or two hind limbs); 3, paraplegia (complete paralysis of two hind limbs); 4, paraplegia with forelimb weakness or paralysis; 5, moribund or death. After the onset of EAE, food and water were provided on the cage floor. Mononuclear cells were prepared from the CNS (brain and spinal cord) of EAE-induced mice as described and analyzed by flow cytometry.

### In vivo infections

For LM-OVA infection, LM-OVA was grown in TSB medium to an OD_600_ of ~0.25, diluted in PBS and injected (8 × 10^3^ CFU) in a volume of 0.2 ml per mouse *i.v*.. After 5 days, the mice were analyzed. For the LM-CFU colony forming assay, 100-μl samples were added to TSB solid medium and incubated overnight at 37 °C. For LCMV clone 13 infection, 1 × 10^6^ PFU were diluted in PBS and injected *i.p*. into mice. LCMV titers were determined by extracting mRNA from extracted organs, and performing qPCR with virus-specific primers.

### Immune cell isolation

Skin: Mouse ear or back skin was incubated in 0.25% Trypsin-EDTA (25-050-CI, Corning) for 45 min, and epidermis and dermis layers were separated with forceps. Separated skin layers were chopped into small pieces and incubated in digestion buffer (1 mg/mL Collagenase P, 0.1 mg/mL DNase I, and 1 mg/mL Dispase II) for 45 to 90 min, respectively, to obtain single-cell suspension.

Lymphoid organs: To harvest immune cells from lymphoid tissue, organs were minced, ground up, and passed through a 70-μm nylon mesh. Erythrocytes were removed using ACK lysis buffer (150 mM ammonium chloride, 10 mM potassium bicarbonate, and 0.1 mM EDTA). The cells were counted using Beckman Coulter CytoFlex. Before sorting, DCs were enriched with CD11c microbeads (Miltenyi Biotec). For peripheral tissues, organs were digested in collagenase D (Roche) and DNase I (Sigma-Aldrich) for 1 h at 37 °C with stirring in PBS. After enzymatic treatment, tissues were further dissociated over a 70μm nylon cell strainer. For isolation of lymphocytes single-cell suspensions were then separated using a 44%/67% Percoll (Sigma) density gradient.

Peritoneal cavity macrophages: Peritoneal exudate cells (PECs) were obtained by lavaging the peritoneal cavity twice with 5 mL ice-cold PBS. PECs were collected in a 15-ml tube, and centrifuge at 500 × *g* for 5 min. Peritoneal macrophages were purified from PECs (>90%) with short incubating on tissue-culture plates and washing away unattached cells. These cells were stimulated by various agonists as indicated.

### In vitro stimulation and flow cytometry

In case of intracellular cytokine staining, 10ug/ml brefeldin A (BFA, eBiosciences) was added with TLR ligands (1 µg/ml R848, 1 µM CpG A, 1 µM CpG B, 1 µg/mL Pam3CSK4, 100 ng/mL LPS, 500 ng/mL Poly(I:C) for 4–16 h before staining with the intracellular staining kit (Cat#88-8824-00, Thermofisher). In case of ELISA, supernatants were collected 4–16 h after TLR stimulations. The following antibodies were all used at dilution of 1:400 (unless otherwise indicated):

eBioscience CD45FITCClone:30-F11Catalogue #:11-0451-82

eBioscience CD45PEClone:30-F11Catalogue #:12-0451-82

eBioscience CD45APCClone:30-F11Catalogue #:17-0451-82

eBioscience CD45.1PE-Cyanine7Clone:A20Catalogue #:25-0453-82

eBioscience CD45.1PBClone:A20Catalogue #:48-0453-82

eBioscience CD45.2APC-Cyanine7Clone:104Catalogue #:47-0454-82

Biolegend XCR1PerCP/Cyanine5.5Clone: ZETCatalogue #:148207

eBioscience CD172aPEClone:P84Catalogue #:12-1721-82

eBioscience CD172aPE-Cyanine7Clone:P84Catalogue #:25-1721-82

eBioscience MHC Class IIPEClone:M5/114.15.2Catalogue #:12-5321-82

eBioscience MHC Class IIPE-Cyanine7Clone:M5/114.15.2Catalogue #: 25-5321-82

eBioscience MHC Class IIPBClone:M5/114.15.2Catalogue #:48-5321-82

eBioscience CD11cPEClone:N418Catalogue #:12-0114-82

eBioscience CD11cAPC-Cyanine7Clone:N418Catalogue #: 47-0114-82

eBioscience CD8aPerCP/Cyanine5.5Clone:53-6.7Catalogue #:35-0081-82

eBioscience CD11bPerCP-Cyanine5.5Clone:M1/70Catalogue #:45-0112-82

eBioscience CD11bFITCClone:M1/70Catalogue #:11-0112-82

eBioscience CD4APC-Cyanine7Clone:RM4-5Catalogue #: 47-0042-82

eBioscience CD4eFluor 450Clone:RM4-5Catalogue #:48-0042-82

eBioscience BST2APCClone:eBio927Catalogue #:17-3172-82

eBioscience BST2FITCClone:eBio927Catalogue #: 11-3172-82

eBioscience SIGLEC HAPCClone:eBio440cCatalogue #:17-0333-82

eBioscience SIGLEC HFITCClone:eBio440cCatalogue #:11-0333-82

eBioscience B220eFluor 450Clone:RA3-6B2Catalogue #:48-0452-82

eBioscience TNF alpha PE-Cyanine7 Clone:MP6-XT22Catalogue #:25-7321-82

eBioscience CD24PEClone:M1/69Catalogue #:12-0242-82

eBioscience F4/80PEClone:BM8Catalogue #:12-4801-82

eBioscience F4/80eFluor 450Clone:BM8Catalogue #:MF48004-3

eBioscience IgDAPC-eFluor 780Clone:11-26c(11-26)Catalog #:47-5993-82

eBioscience IgM PEClone:II/41Catalogue #:12-5790-82

eBioscience IgM Super Bright 600Clone:II/41Catalogue #:63-5790-82

eBioscience CD93APCClone:AA4.1Catalogue #:17-5892-82

Biolegend CD5APCClone:53-7.3Catalogue #:100626

Biolegend Ly-6CAlexa Fluor® 488Clone:HK1.4Catalogue #:128022

eBioscience Ly-6GeFluor 450Clone:1A8-Ly6gCatalogue #:48-9668-82

eBioscience CD3e APCClone:145-2C11Catalogue #:MA1-10186

eBioscience CD3e eFluor 450Clone:145-2C11Catalogue #:48-0031-82

eBioscience TCR-γδAPCClone:eBioGL3Catalogue #:17-5711-82

eBioscience TCR-γδPE-Cyanine7 Clone:eBioGL3 Catalog #:25-5711-82

eBioscience TCR βPEClone:H57-597Catalogue #:12-5961-82

eBioscience TCR βAPC-eFluor 780Clone:H57-597Catalogue #:47-5961-82

eBioscience IFN-γPEClone:XMG1.2Catalogue #:12-7311-82

eBioscience IFN-γPerCP/Cyanine5.5Clone:XMG1.2Catalogue #:45-7311-82

eBioscience IL-17AFITCClone:eBio17B7Catalogue #:11-7177-81

Biolegend CD80FITCClone:16-10A1Catalogue #:104706

Biolegend CD80PEClone:16-10A1Catalogue #:104708

MBL Ly49QPEClone:2E/6Catalogue #: D160-4

eBioscience CD86FITCClone:GL1 Catalog #:11-0862-82

eBioscience CD86PEClone:GL1 Catalog #:12-0862-82

eBioscience CD199PerCP-eFluor 710Clone:eBioCW-1.2Catalogue #:46-1991-82

eBioscience CD64PerCP-eFluor 710Clone:X54-5/7.1Catalogue #:46-0641-82

PBLassay IFNaFITCClone:RMMA-1Catalogue #:22100-3

Biolegend CCR2PE-Cyanine7Clone:QA18A56Catalogue #:160108

eBioscience Ly-6A/E (Sca-1)APCClone:D7Catalogue #: 17-5981-82

eBioscience Ly-6A/E (Sca-1)PE-Cyanine7Clone:D7Catalogue #:25-5981-82

eBioscience IL-12p40eFluor 660Clone:C17.8Catalogue #:50-7123-82

Invivogen IL-6N.AClone:10F9Catalogue #:mabg-mil6-3

Flow cytometry was performed on Beckman Coulter CytoFlex and analyzed using FlowJo software (Tree Star, version X). MFI were calculated by genomic mean in FlowJo. Cell-sorting experiments were conducted on a BD FACSAria III. Staining was performed at 4 °C in the presence of Fc block (Clone 2.4G2; BD) and FACS buffer (PBS, 0.5% BSA, 2 mM EDTA, 0.1% sodium azide).

### Primary cell culture

Mice were sacrificed by cervical dislocation and removal of both femurs and tibias. After removing the muscle and fat with a pair of scissors, the femurs and tibias were put in 5 ml RPMI 1640 (10% FCS and PS) on ice. The ends of the bone were cut with a sharp pair of scissors in the tissue-culture hood and then the BM were flushed out with a 10-ml syringe (25-G needle) and RPMI 1640 media (10% FCS, PS). BM cells were collected in a 15-ml tube, and centrifuge at 500 × *g* for 5 min.

#### Bone marrow-derived macrophages (BMDM)

5 × 10^6^ bone marrow cells per well were cultured in non-pyrogenic sterilized 90 mm petri dishes in RP10 (RPMI supplemented with glutamine, penicillin, streptomycin; Invitrogen), 10% FBS and 30% supernatants of L929 mouse fibroblasts (ATCC ^®^ CRL-6364™) as conditioned medium providing macrophage colony stimulating factor (M-CSF). Half of media was replenished on day 3 and day 6, and harvested on day 7. To harvest macrophages, cultured cells were washed once with ice-cold PBS, and then incubated in PBS containing 20 mM EDTA and 20% FCS at 37 °C for 5 min. BMDMs were gated as live^+^ CD45^+^ CD11b^+^ F4/80^+^ cells.

#### FL3TL-pDCs

4 × 10^6^ bone marrow cells per well were cultured in tissue-culture-treated 6-well plates in 4 ml of RP10 (RPMI 1640 supplemented with glutamine, penicillin, streptomycin, 2-mercaptoethanol [all from Invitrogen]), 10% heat-inactivated fetal calf serum (Source BioSciences), and 30% supernatants of Flt3L-B16 cell line (ATCC® CRL-6475™ retrovirally transduced in house with murine *Flt3l*) as conditioned medium providing Flt3L. FL3TL-pDCs were gated as live^+^ CD11c^+^ B220^+^ SiglecH^+^ cells. These cells were harvested on day 7.

#### BMDC

4 × 10^6^ bone marrow cells per well were cultured in tissue-culture-treated 6-well plates in 4 ml of RP10 (RPMI 1640 supplemented with glutamine, penicillin, streptomycin, 2-mercaptoethanol [all from Invitrogen]), 10% heat-inactivated fetal calf serum (Source BioSciences), IL-4(5 ng/ml, Peprotech) and GM-CSF (20 ng/ml, Peprotech). Half of the medium was removed at day 2, and new medium supplemented with GM-CSF (40 ng/ml), IL-4(10 ng/ml) which were pre-warmed at 37 °C was added. The culture medium was entirely discarded at day 3 and replaced with fresh warmed medium containing GM-CSF (20 ng/ml) and IL-4(5 ng/ml). BMDCs were gated as live^+^ CD11b^+^ MHCII^+^ CD11c^+^ cells. These cells were harvested on day 6.

#### pDCs/BMDM co-culture

5 × 10^4^ Flt3L-pDCs were plated on 96-well plates with varying numbers, and pre-condition with 1 µg/mL R848 for 16 h. Varying ratio of BMDMs were added in wells containing Flt3L-pDCs as indicated in the figure legends. These co-cultured cells were stimulated for 4 h with 1 µg/mL R848 in presence of 10 µg/mL Brefeldin A for intracellular cytokines stain. pDC supernatant with BMDM: BMDM were stimulated with 1 µg/mL R848 in presence of 10 µg/mL Brefeldin A in medium containing various concentration of supernatant from Flt3L-pDCs which were stimulated for 16 h with indicated ligands.

#### Cytokine/antibodies additions

rIL6: BMDM were treated with IL-6 (Cat#50136-MNAE, Sino Biological) for along with 1 µg/mL R848 for 4 h. IFN-I: BMDMs were pre-conditioned with IFN-alpha4 (Cat#50672-M08H, Sino Biological) for 16, 24, or 48 h, and then activated with R848(1 µg/ml) for 4 h. The supernatant was harvested and stored in −80 °C refrigerator for subsequent ELISA measurement. Anti-IL-6: 3.5 × 10^5^ sorted (purity>99%) Flt3L-pDC activated with 1 µg/ml R848 containing in 1.4 ml RP10 for 24 h. Then Flt3L-pDC supernatants were pre-incubated with 1 µg/ml anti-IL6 mIgG (Cat#mabg-mIL6-3, Invivogen) for 30 min. 3 × 10^5^ BMDMs were co-cultured with 50% Flt3l-pDC supernatant or anti-IL6 pre-treated Flt3L-pDC supernatant in the presence of 1 µg/ml R848 and 10 µg/ml brefeldin A for 4 h, and were analyzed by intracellular staining with FACS.

### Molecular cloning

Mouse cDNA of *Zc3h12a* (NM_153159.2), *Zc3h12c* (NM_001162921.2), *Il6* (NM_031168.2), *Tnf* (NM_013693.3), *Nfkbiz*(NM_030612) were reverse transcribed with HiScript II 1st Strand cDNA Synthesis Kit (Vazyme, Cat#R212-02) from the mRNA of BMDM after treated with R848 for 4 h, then all were cloned into pTOPO vector (GenStar,Cat#T182-20). Point mutations of the gene encoding *Zc3h12a* (D141N, C316R) and *Zc3h12c* (D271A, D271N, D271A, D356A, S372A, D374A, D378A, C436R), and human SNP rs4561177 were made with Mut Express II Fast Mutagenesis Kit V2 (Vazyme, Cat#C214-02). Myc-tag and Flag-tag were added to the N-terminal of CDS of *Zc3h12a* and *Zc3h12c* respectively. The CDSs of N-Myc-*Zc3h12a* and N-Flag-*Zc3h12c* were ligated to the vector pWPI or pLVX for expression. Full length of 3’UTR of *Il6* (1-427), *Tnf* (1-764), β-*actin* (1-683), *Zc3h12a* (1-856), *Zc3h12c* (1-3794), *ccr7* (1-762), *Nfkbiz* (1-1294) were insert into the pGL3 vector at the site of XbaI site. These CDS + 3′UTR was inserted into pLVX-puro (Clontech, Cat # 632164) or pWPI (Addgene, Cat#12254), along with pGL3 vector.

### Luciferase reporter assay

293T (ATCC^®^ CRL-3216™) cells were transfected with the firefly/Renilla DuoLuc-Luciferase reporter vector and an expression plasmid for as indicated via Exfect 2000 Transfection Reagent (Vazyme, Cat# T202-02). 48 h later, the cells were lysed, and firefly and Renilla activity was determined according to the manufacturer’s protocol of the Dual Luciferase Reporter Assay Kit (Vazyme, Cat#DL101-01). Firefly activity was normalized to the Renilla activity.

### Protein degradation assay

For Regnase-1 degradation assay, 2.5 × 10^5^ 293T cells were plated into 24-well cell culture plate for 24 h. 0.06 pM *Zc3h12c* containing pLVX-vector were co-transfected with 0.01pM *Zc3h12a*-CDS or *Zc3h12a* -CDS-3’ UTR containing pWPI-plasmid into 293T cell in the presence of PEI. After 48 h, whole cell extracts were prepared with low-salt lysis buffer (50 mM HEPES, 150 mM NaCl, 1 mM EDTA, 10% glycerol, 1.5 mM MgCl, and 1% Triton X-100) after transfection. The samples were denatured with 5× loading buffer (Genstar, E153-05) for 5 min at 95 °C. Total cell lysates (60 µg) were running on SDS-PAGE with 4–12% gradient gel and then transferred. Proteins separated by SDS-PAGE were transferred to polyvinylidene fluoride (PVDF) membranes (Bio-Rad). The membranes were blocked by 5% dried milk at 4 °C overnight, and then incubated with HRP-conjugated antibodies for 1 h at room temperature. The following antibody were used: Mouse monoclonal anti-Flag (M2) peroxidase (HRP) (Sigma-Aldrich, Cat#A8592, 1:1000), Mouse monoclonal anti-c-Myc-HRP (Roche Applied Science, Cat#11814150001, 1:1500), Mouse monoclonal anti-β-actin (Sigma-Aldrich, Cat# A3854, 1:3000). After washing with PBST, Immobilon Western Chemiluminescent HRP Substrate (Millipore) was used for secondary protein detection. The bands were visualized by chemical composition using ChemiDoc Touch (BIO-RAD). The uncropped and unprocessed scans of SDS-PAGE is provided in the source data file.

For IL-6 degradation assay, 2.5 × 10^5^ 293T cells were plated into 24-well cell culture plate for 24 h. 0.06 pM *Zc3h12a* or *Zc3h12c* containing pLVX-vector were co-transfected with 0.01pM IL6-CDS or IL6-CDS-3UTR containing pWPI-plasmid into 293T cell in the presence of PEI. After 48 h, supernatants were collected for ELISA, and mRNAs were extracted for qPCR.

For TNF degradation assay, 0.06 pM *Zc3h12a* or *Zc3h12c* containing pLVX-vector were co-transfected with 0.03 pM pWPI-TNF-CDS or TNF-CDS-3UTR containing plasmid. After 48 h, supernatants were collected for ELISA.

### RNA extraction and quantitative PCR (qPCR)

TRIzol reagent (Genstar, Cat#P118-05) and chloroform were added to homogenize single cells, followed by RNA precipitation, washing and resuspension following the manufacturer’s protocol. The extracted RNA was used for reverse transcription according to the manufacturer’s protocol (HiScript III RT SuperMix for qPCR, Vazyme, R323-01). Quantitative RT-PCR analysis was performed with SYBR Select Master Mix (Genstar, Cat#A301-10) using StepOne Plus (Life Sciences). All data were normalized to *β-Actin* expression. qPCR primer sequences are listed in Supplementary Table 1.

### ELISA

Samples were placed on pre-coated plates with anti-mouse TNFα (Cat # 88-7324-88, Thermofisher), IL-6 (Catalog # 88-7064-88 Thermofisher), IFNα (Cat# # BMS6027, Invitrogen), and performed according to manufacturer’s instructions.

### Statistical analysis

Statistical analysis was performed in GraphPad Prism 6 software. For reanalysis of human microarray samples, two-tailed Wilcoxon Matched-pair Signed Rank Test was used. For murine samples, comparisons between two groups were performed by two-tailed Student’s *t* test. For human PBMC samples, two-tailed Mann–Whitney test was used. Two human samples (one each from CD4^+^ T and macrophages groups) were excluded as outliers, due to their Z-scores exceeding ±3. A *P* value of 0.05 or less was considered significant. The survive curves were calculated using Log-rank (Mantel-Cox) test.

### Reporting summary

Further information on research design is available in the Nature Research Reporting Summary linked to this article.

## Supplementary information

Supplementary Information

Reporting Summary

## Data Availability

No novel datasets are generated in this study. Data that support the findings of this study are reanalysis of publicly available datasets. Patient biopsy datasets are available at Gene Expression Omnibus (GEO) repository (https://www.ncbi.nlm.nih.gov/gdsin) with accession codes of GDS3539; GDS4600; GSE53552. RNA-seq datasets are available at The Immunological Genome Project (ImmGen) (https://www.immgen.org) with the search term “Zc3h12c” (http://rstats.immgen.org/Skyline/skyline.html). SNP analysis is available at The Genotype-Tissue Expression (GTEx) (https://www.gtexportal.org/) with the search term of “rs4561177” (https://www.gtexportal.org/home/snp/rs4561177). Source data are provided with this paper.

## Source data

Source Data
