# Supplementary material for: The RNase MCPIP3 promotes skin inflammation by orchestrating myeloid cytokine response
Source: Nat Commun. 2021 Jul 2;12:4105. doi: 10.1038/s41467-021-24352-w (PMC8253787; doi:10.1038/s41467-021-24352-w)
Supplement: Supplementary file 1 — Supplementary Information [file 41467_2021_24352_MOESM1_ESM.pdf]

List of Supplementary Information

Supplementary Fig. 1 Generation of MCPIP3-deficient mice
Supplementary Fig. 2 MCPIP3 is dispensable for immune cell development
Supplementary Fig. 3 MCPIP3 does not regulate classic M1/M2 functions
Supplementary Fig. 4 MCPIP3 is dispensable for IFN-α and TNF production by DCs
Supplementary Fig. 5 The role of MCPIP3 in various immune subsets involved in psoriasis
Supplementary Fig. 6 Screening of transcriptional regulators in MCPIP3-deficient macrophages
Supplementary Fig. 7 Screening of transcriptional regulators in MCPIP3-deficient pDCs
Supplementary Fig. 8 Flow cytometry gating strategies
Supplementary Table 1: Primer sequences
Source Data: Raw data for all figures

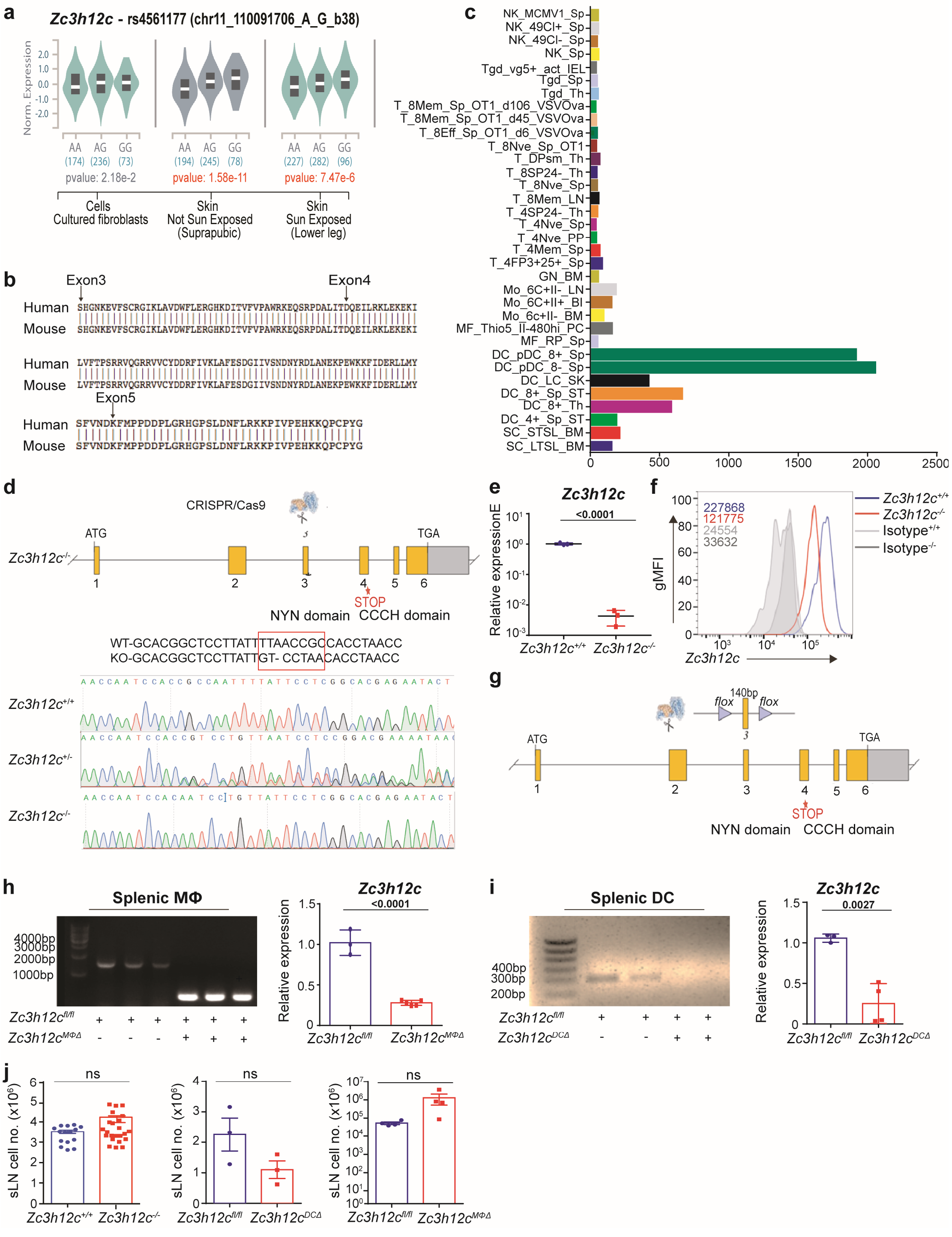

Supplementary Fig. 1 Generation of MCPIP3-deficient mice

a eQTL analysis of SNP (rs4561177) using GTEx at www.gtexportal.org.

b Amino acid sequence comparison of MCPIP3 Exon 3-5 between humans and mice.

c Expression of *Zc3h12c* mRNA (in arbitrary units) in the indicated populations reanalyzed from the IMMGEN gene microarray.

d Schematics for the generation of *Zc3h12c*$^{-/-}$ mice.

e *Zc3h12c* mRNA expression from sorted bone marrow pDCs from *Zc3h12c*$^{+/+}$/*Zc3h12c*$^{-/-}$ mice, measured by qPCR (n=3 mice per group).

f MCPIP3 protein expression from bone marrow pDCs from *Zc3h12c*$^{+/+}$/*Zc3h12c*$^{-/-}$ mice,

measured by anti-MCPIP3 polyclonal antibodies (n=3 pooled mice per group).

g Schematics for the generation of *Zc3h12c*$^{fl/fl}$ mice.

h Verification of *Zc3h12c*^{MΦΔ} mice. Splenic $CD11b^+$ $F4/80^+$ macrophages from *Zc3h12c*$^{fl/fl}$/ *Zc3h12c*^{MΦΔ} were FACS-sorted, and checked for deletion with PCR (left) and qPCR(right) (n=3 mice per group).

i Verification of *Zc3h12c*^{DCΔ} mice. Splenic $CD11c^+$ cells from *Zc3h12c*$^{fl/fl}$/ *Zc3h12c*^{DCΔ} were FACS-sorted, and checked for deletion with PCR (left) and qPCR(right) (n=3 mice per group).

j Skin-draining (popliteal, axillary, and brachial) lymph node cell numbers of *Zc3h12c*$^{+/+}$/*Zc3h12c*$^{-/-}$, *Zc3h12c*$^{fl/fl}$/ *Zc3h12c*^{DCΔ}, *Zc3h12c*$^{fl/fl}$/ *Zc3h12c*^{MΦΔ} mice (~12 weeks) (n=18 mice for *Zc3h12c*$^{+/+}$; n=23 mice for *Zc3h12c*$^{-/-}$; n=3 for *Zc3h12c*$^{fl/fl}$/ *Zc3h12c*^{DCΔ}; n=5 for *Zc3h12c*$^{fl/fl}$/ *Zc3h12c*^{MΦΔ}).

Source data are provided as a Source Data file (two-tailed Student's t-test). ns= not significant. Data are representative of at least two independent experiments (mean±S.D.).

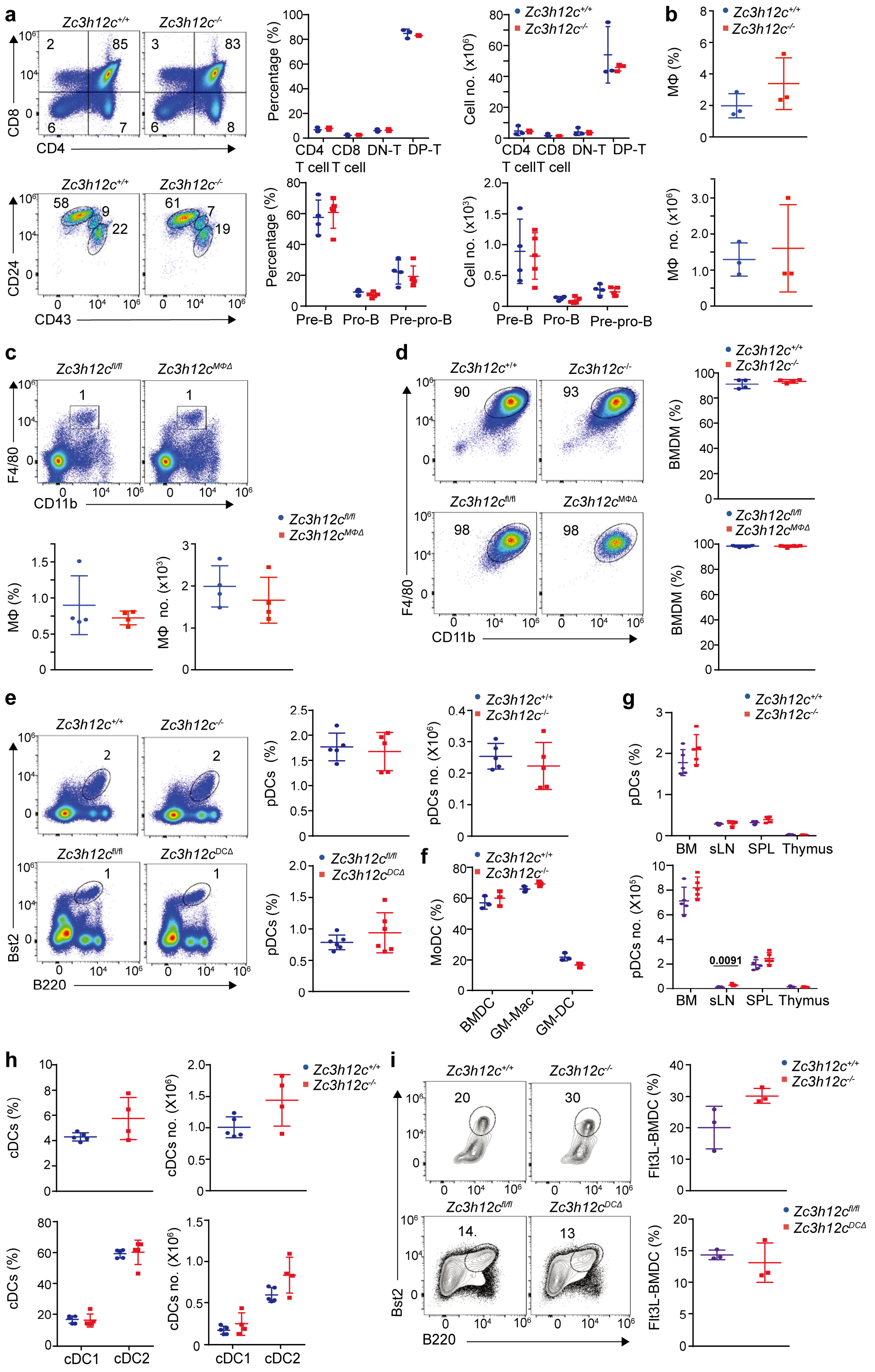

Supplementary Fig. 2 MCPIP3 is dispensable for immune cell development

a Distribution of T and B cell subsets in *Zc3h12c*$^{+/+}$/*Zc3h12c*$^{-/-}$ mice (n=4 mice per group).

b Distribution of splenic macrophages in *Zc3h12c*$^{+/+}$/*Zc3h12c*$^{-/-}$ mice (n=3 mice per group).

c Distribution of splenic macrophages in *Zc3h12c*$^{fl/fl}$/ *Zc3h12c*$^{M\Phi\Delta}$ mice (n=4 mice per group).

d Percentages of macrophages from BMDMs in *Zc3h12c*$^{+/+}$/*Zc3h12c*$^{-/-}$ and *Zc3h12c*$^{fl/fl}$/ *Zc3h12c*$^{M\Phi\Delta}$ mice (n=3 mice per group).

e Distribution of bone marrow pDCs in *Zc3h12c*$^{+/+}$/*Zc3h12c*$^{-/-}$ and *Zc3h12c*$^{fl/fl}$/ *Zc3h12c*$^{DC\Delta}$ mice (n=5 mice per group).

f Percentages of *Zc3h12c*$^{+/+}$/*Zc3h12c*$^{-/-}$ BMDC and subsets (n=3 mice per group).

g Distribution of pDCs in different immune organs of *Zc3h12c*$^{+/+}$/*Zc3h12c*$^{-/-}$ mice (n=5 mice per group).

h Distribution of splenic cDCs in *Zc3h12c*$^{+/+}$/*Zc3h12c*$^{-/-}$ mice (n=4 mice per group).

i Percentages of FL3TL-pDCs in *Zc3h12c*$^{+/+}$/*Zc3h12c*$^{-/-}$ and *Zc3h12c*$^{fl/fl}$/*Zc3h12c*$^{DC\Delta}$ mice (n=3 mice per group).

Source data are provided as a Source Data file (two-tailed Student's t-test). Data are representative of at least two independent experiments (mean±S.D.).

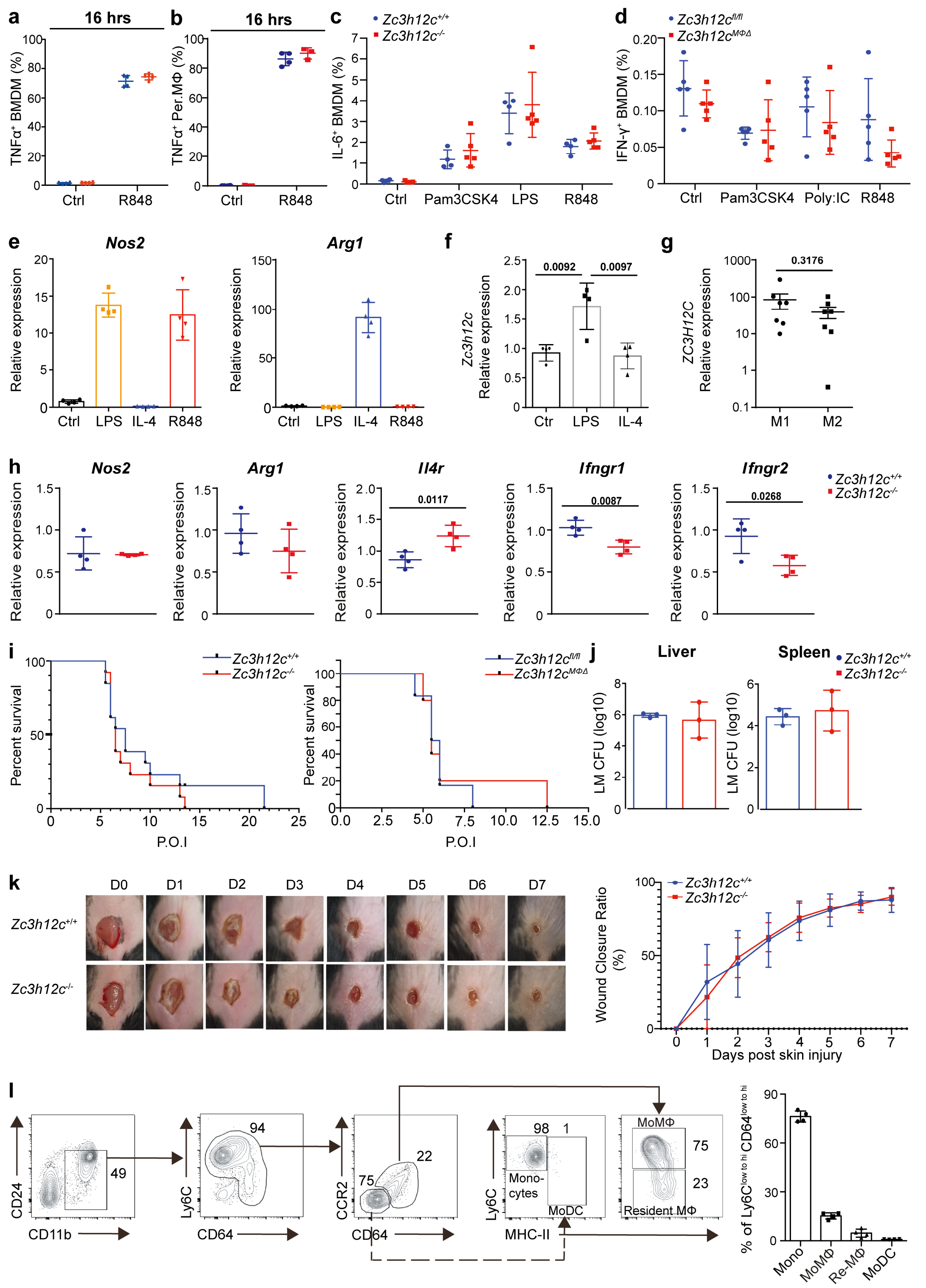

Supplementary Fig. 3 MCPIP3 does not regulate classic M1/M2 functions

a TNF production by activated *Zc3h12c*$^{+/+}$/*Zc3h12c*$^{-/-}$ BMDMs was measured by intracellular staining after 16 hours (n=4 mice per group).

b TNF production by activated *Zc3h12c*$^{+/+}$/*Zc3h12c*$^{-/-}$ Per.MΦ was measured by intracellular staining after 16 hours (n=4 mice per group).

c IL-6 production (intracellular staining) by *Zc3h12c*$^{+/+}$/*Zc3h12c*$^{-/-}$ BMDMs stimulated in vitro with indicated ligands (n=5 mice per group).

d IFN-γ production (intracellular staining) by *Zc3h12c*$^{+/+}$/*Zc3h12c*$^{-/-}$ BMDMs stimulated in vitro with indicated ligands (n=5 mice per group).

e *Nos2* and *Arg1* expression in M1/M2/R848 conditioned *Zc3h12c*$^{+/+}$ BMDMs (n=4 mice per group).

f *Zc3h12c* expression in M1/M2 conditioned WT BMDMs (n=4 mice per group).

g *ZC3H12C* expression in M1/M2 conditioned healthy human PMBC monocyte-derived macrophages (n=7 individuals per group).

h Signature M1/M2 genes expression in R848-conditioned *Zc3h12c*$^{+/+}$/*Zc3h12c*$^{-/-}$ BMDMs (n=4 mice per group).

i Survival curve of LPS-induced sepsis in *Zc3h12c*$^{+/+}$/*Zc3h12c*$^{-/-}$ and *Zc3h12c*$^{fl/fl}$/ *Zc3h12c* ^{MΦΔ} mice (n=10 mice per group).

j Bacterial load (CFU) of liver and spleen from *Zc3h12c*$^{+/+}$/*Zc3h12c*$^{-/-}$ mice infected with *Listeria monocytogenes* (n=3 mice per group).

k Wound closure rate of *Zc3h12c*$^{+/+}$/*Zc3h12c*$^{-/-}$ mice (n=5 mice per group).

l Distribution of myeloid cells from IMQ-treated back-skin of WT mice at day 3. Monocytes ($CD11b^{+}$ $CD24^{low}$, $Ly6c^{low\ to\ hi}$ $CD64^{low\ to\ hi}$, $F4/80^{-}$ $CCR2^{-}$, $Ly6C^{+}$ $MHCII^{-}$), MoDC ($CD11b^{+}$ $CD24^{low}$, $Ly6c^{low\ to\ hi}$ $CD64^{low\ to\ hi}$, $F4/80^{-}$ $CCR2^{-}$, $Ly6c^{+}$ $MHCII^{+}$), MoMΦ ($CD11b^{+}$ $CD24^{low}$, $Ly6c^{low\ to\ hi}$ $CD64^{low\ to\ hi}$, $F4/80^{+}$ $CCR2^{+}$, $Ly6c^{hi}$ $MHCII^{+}$), resident MΦ ($CD11b^{+}$ $CD24^{low}$, $Ly6c^{low\ to\ hi}$ $CD64^{low\ to\ hi}$, $F4/80^{+}$ $CCR2^{+}$, $Ly6c^{low}$ $MHCII^{+}$) were calculated as percentages of live $CD45^{+}$ $CD11b^{+}$ cells (n=4 mice per group).

Data are representative of at least two independent experiments (mean±S.D.) Source data are provided as a Source Data file (two-tailed Student's t-test for murine samples; two-tailed Mann Whitney test for human samples; Log-rank (Mantel-Cox) test for survival curves).

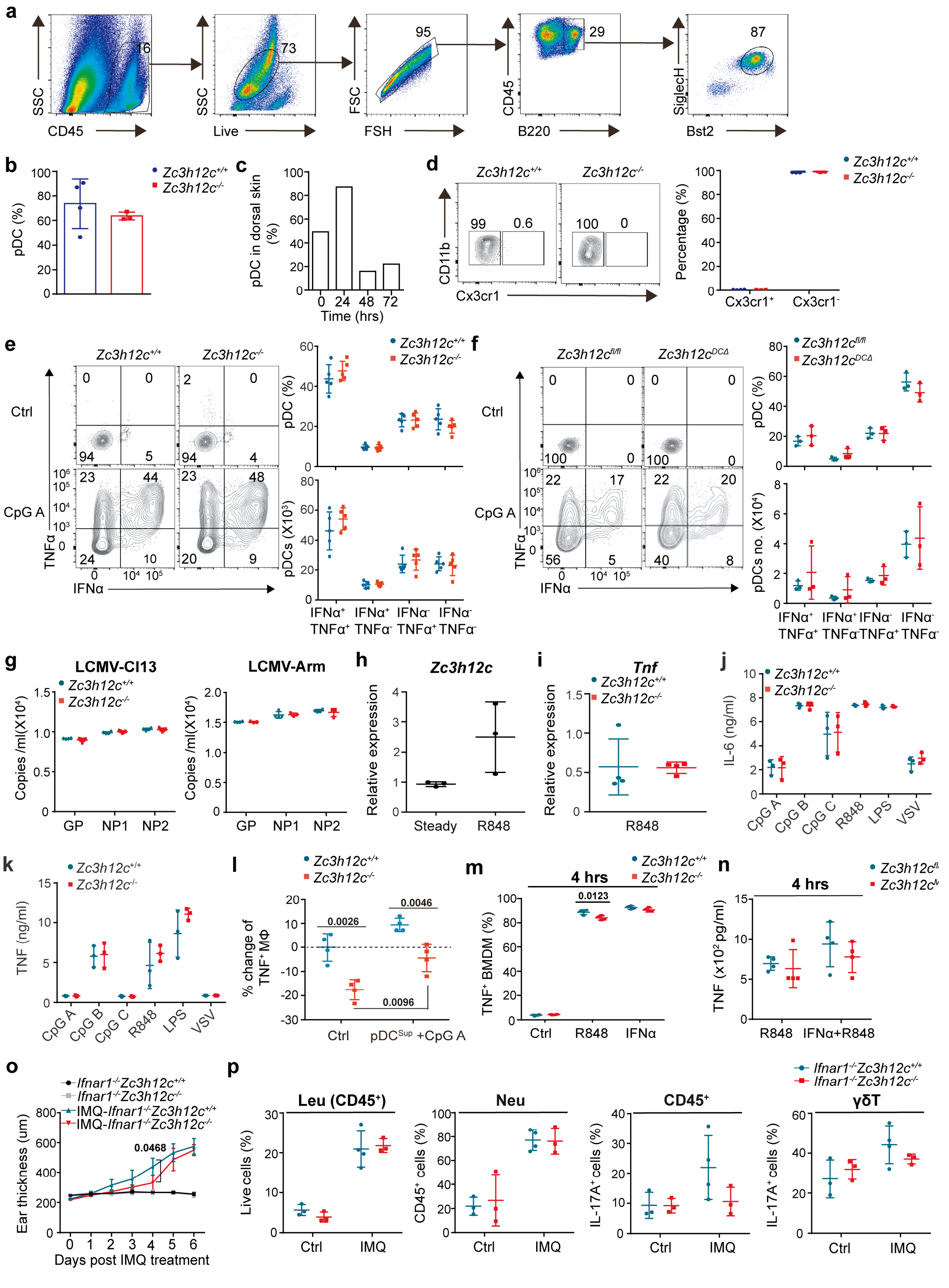

Supplementary Fig. 4 MCPIP3 is dispensable for IFN-α and TNF production by DCs

a FACS gating strategy to identify pDCs in IMQ-treated skin on day 1.

b pDC percentages from IMQ-treated back-skin of *Zc3h12c*$^{+/+}$/*Zc3h12c*$^{-/-}$ mice on day 1 (n=5 mice per group).

c pDC percentages from WT IMQ-treated back-skin on indicated days (n= 1 mice per group)

d CX3CR1 expression of pDCs in IMQ-treated skin of *Zc3h12c*$^{+/+}$/*Zc3h12c*$^{-/-}$ mice at day 1 (n=3 mice per group).

e IFN-α and TNF production (measured by intracellular staining) by BM *Zc3h12c*$^{+/+}$/*Zc3h12c*$^{-/-}$ pDCs stimulated in vitro with CpG A and R848 (n=5 mice per group).

f IFN-α and TNF production (measured by intracellular staining) by BM *Zc3h12c*$^{fl/fl}$/ *Zc3h12c*^{DCΔ} pDCs stimulated in vitro with CpG A (n=5 mice per group).

g Viral titer of *Zc3h12c*$^{+/+}$/*Zc3h12c*$^{-/-}$ mice infected with LCMV-Armstrong or clone 13 was measured by qPCR (n=3 mice per group).

h *Zc3h12c* expression in R848-activated sorted splenic *Zc3h12c*$^{fl/fl}$/ *Zc3h12c*^{DCΔ} cDC2s was measured by qPCR (n=4 mice per group)

i *Tnf* expression in sorted splenic *Zc3h12c*$^{fl/fl}$/ *Zc3h12c*^{DCΔ} cDC2s was measured by qPCR after 24 hours of activation with R848 (n=4 mice per group)

j IL-6 secretion by *Zc3h12c*$^{+/+}$/*Zc3h12c*$^{-/-}$ BMDC (moDC) were measured by ELISA (n=4 mice per group).

k TNF secretion by *Zc3h12c*$^{+/+}$/*Zc3h12c*$^{-/-}$ BMDC (moDC) were measured by ELISA (n=4 mice per group).

l Sorted WT FL3TL-pDCs were treated with CpG A for 24 hours, and their supernatants (pDC^{sup}) were added to *Zc3h12c*$^{+/+}$/*Zc3h12c*$^{-/-}$ BMDMs along with R848. TNF production in macrophages was measured by intracellular staining. Percentage changes was normalized against *Zc3h12c*$^{+/+}$ samples without pDC^{sup} (n=4 mice per group).

m *Zc3h12c*$^{+/+}$/*Zc3h12c*$^{-/-}$ BMDMs were cultured with recombinant (1mg/ml) IFN-α for 48 hours, then treated with R848. TNF production is measured by intracellular staining after 4 hours (n=3 mice per group).

n *Zc3h12c*$^{+/+}$/*Zc3h12c*$^{-/-}$ BMDMs were cultured with recombinant (1mg/ml) IFN-α for 48 hours, then treated with R848. TNF production is measured by ELISA after 4 hours (n=4 mice per group).

o Ear skin thickness of the IMQ-treated *Ifnar1*$^{-/-}$: *Zc3h12c*$^{+/+}$ or *Ifnar1*$^{-/-}$: *Zc3h12c*$^{-/-}$ mice were measured daily (n=4 mice for *Zc3h12c*$^{+/+}$; n=3 mice for *Zc3h12c*$^{-/-}$).

p Infiltrated CD45^{+} or Ly6G^{+} immune cell percentages, IL-17A production by CD45^{+} or γδ T cells at the IMQ-treated *Ifnar1*$^{-/-}$: *Zc3h12c*$^{+/+}$ or *Ifnar1*$^{-/-}$: *Zc3h12c*$^{-/-}$ mice were measured by FACS (n=4 mice for *Zc3h12c*$^{+/+}$; n=3 mice for *Zc3h12c*$^{-/-}$).

Source data are provided as a Source Data file (two-tailed Student's t-test). Data are representative of at least two independent experiments (mean±S.D.)

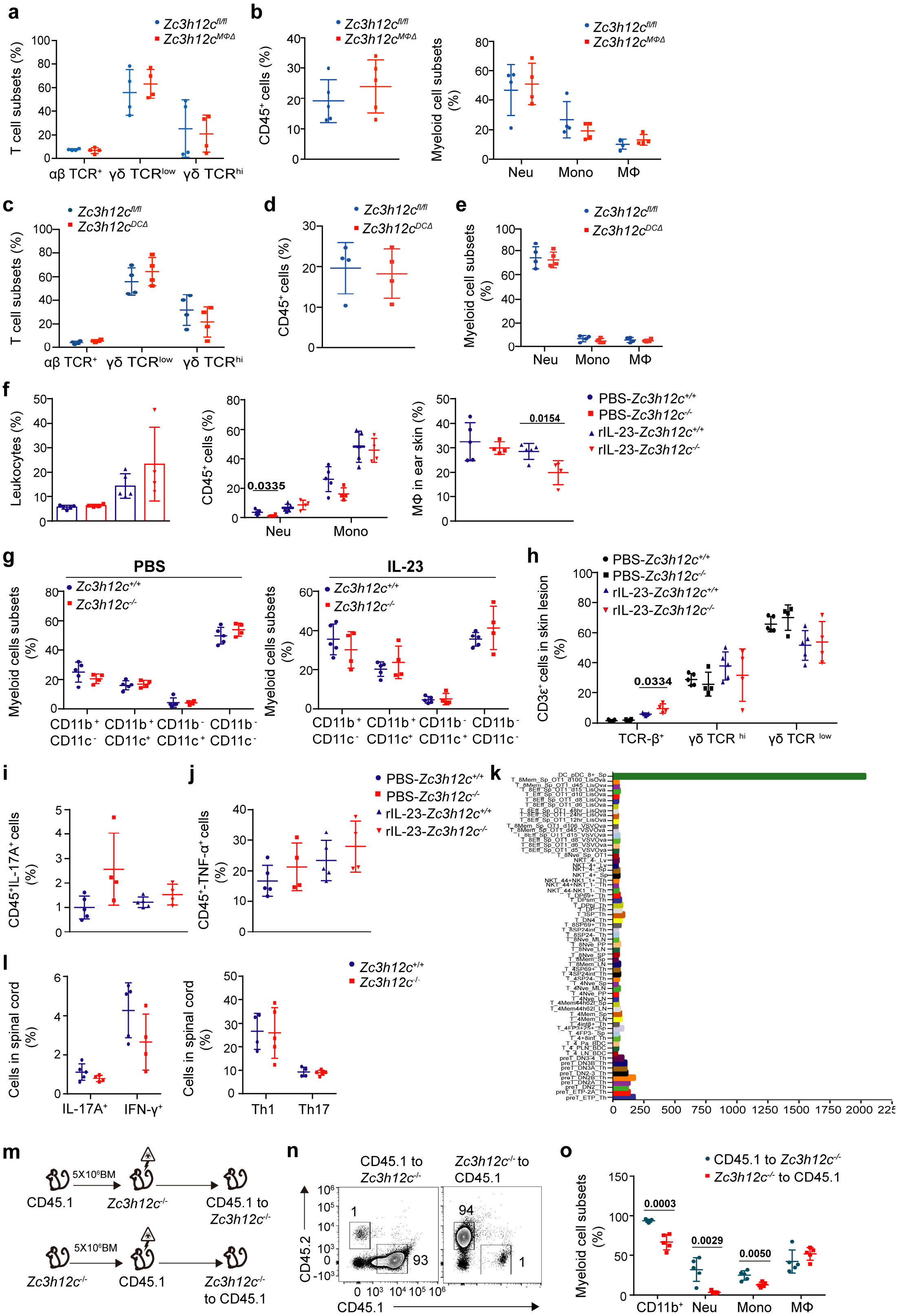

Supplementary Fig. 5 The role of MCPIP3 in various immune subsets involved in psoriasis

a Infiltrated T cell percentages at IMQ-treated skin of *Zc3h12c*$^{fl/fl}$/*Zc3h12c* ^{MΦΔ} mice at D4 (n=5 mice per group).

b Infiltrated immune cell percentages at IMQ-treated skin of *Zc3h12c*$^{fl/fl}$/*Zc3h12c* ^{MΦΔ} mice at

D4 (n=5 mice per group).

c Infiltrated T cell percentages and their IL-17A production at IMQ-treated skin of *Zc3h12c*$^{fl/fl}$/*Zc3h12c*$^{DC\Delta}$ mice at D4 (n=5 mice per group).

d Infiltrated leukocyte percentages at IMQ-treated skin of *Zc3h12c*$^{fl/fl}$/*Zc3h12c*$^{DC\Delta}$ mice at D4 (n=5 mice per group).

e Infiltrated myeloid cell percentages at IMQ-treated skin of *Zc3h12c*$^{fl/fl}$/*Zc3h12c*$^{DC\Delta}$ mice at D4 (n=5 mice per group).

f Infiltrated immune cell percentages at rIL-23-treated skin. Immune cells from IMQ-treated *Zc3h12c*$^{+/+}$/*Zc3h12c*$^{-/-}$ ear-skin were isolated at day 9, and analyzed by flow cytometry (n=5 mice for *Zc3h12c*$^{+/+}$; n=4 mice for *Zc3h12c*$^{-/-}$).

g Infiltrated myeloid cell percentages at rIL-23-treated skin (n=5 mice for *Zc3h12c*$^{+/+}$; n=4 mice for *Zc3h12c*$^{-/-}$).

h Infiltrated T cell percentages at rIL-23-treated skin (n=5 mice for *Zc3h12c*$^{+/+}$; n=4 mice for *Zc3h12c*$^{-/-}$).

i IL-17A production by T cells at rIL-23-treated skin (n=5 mice for *Zc3h12c*$^{+/+}$; n=4 mice for *Zc3h12c*$^{-/-}$).

j TNF production by leukocytes (CD45^{+}) at rIL-23-treated skin (n=5 mice for *Zc3h12c*$^{+/+}$; n=4 mice for *Zc3h12c*$^{-/-}$).

k Expression of *Zc3h12c* mRNA (in arbitrary units) in the indicated T cell populations reanalyzed from the IMMGEN gene microarray.

l IFNγ and IL-17A production by CD4^{+} T cells from spinal cord fluids of EAE-induced *Zc3h12c*$^{+/+}$/*Zc3h12c*$^{-/-}$ mice (n=5 mice for *Zc3h12c*$^{+/+}$; n=4 mice for *Zc3h12c*$^{-/-}$).

m Schematics for the generation of the straight (Zc3h12c$^{-/-}$ BM to CD45.1 host) and reverse (CD45.1 BM to Zc3h12c$^{-/-}$ host) chimeras.

n Verification of the straight/reverse chimeras. FACS plots of host and recipient CD45^{+} cells from spleen ~2 months after irradiation were shown.

o Infiltrated myeloid cell percentages at IMQ-treated skin of chimeras at D4 (n=5 mice per group).

Source data are provided as a Source Data file (two-tailed Student's t-test). Data are representative of at least two independent experiments (mean±S.D.)

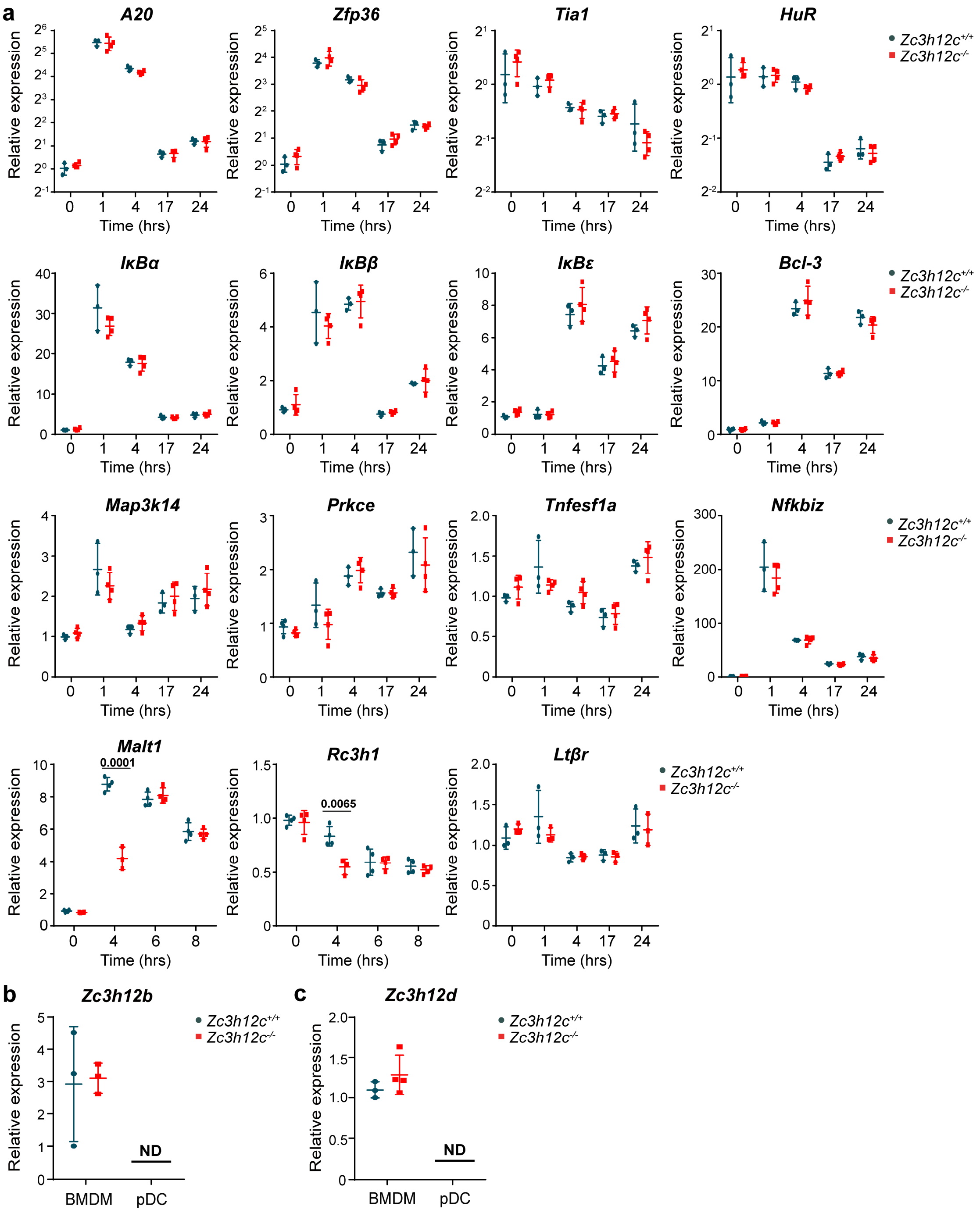

Supplementary Fig. 6 Screening of transcriptional regulators in MCPIP3-deficient macrophages

a mRNA expressions of the indicated genes were measured by qPCR in sorted *Zc3h12c*$^{+/+}$/*Zc3h12c*$^{-/-}$ BMDMs at indicated timepoints after R848 activation (n=3 mice for *Zc3h12c*$^{+/+}$; n=4 mice for *Zc3h12c*$^{-/-}$).

b-c *Zc3h12b* (**b**) or *Zc3h12d* (**c**) expression in sorted *Zc3h12c*$^{+/+}$/*Zc3h12c*$^{-/-}$ BMDMs and FL3TL-pDCs was measured by qPCR (n=3 mice for *Zc3h12c*$^{+/+}$; n=4 mice for *Zc3h12c*$^{-/-}$).

Source data are provided as a Source Data file (two-tailed Student's t-test). ND= not detected. Data are representative of at least two independent experiments (mean±S.D.)

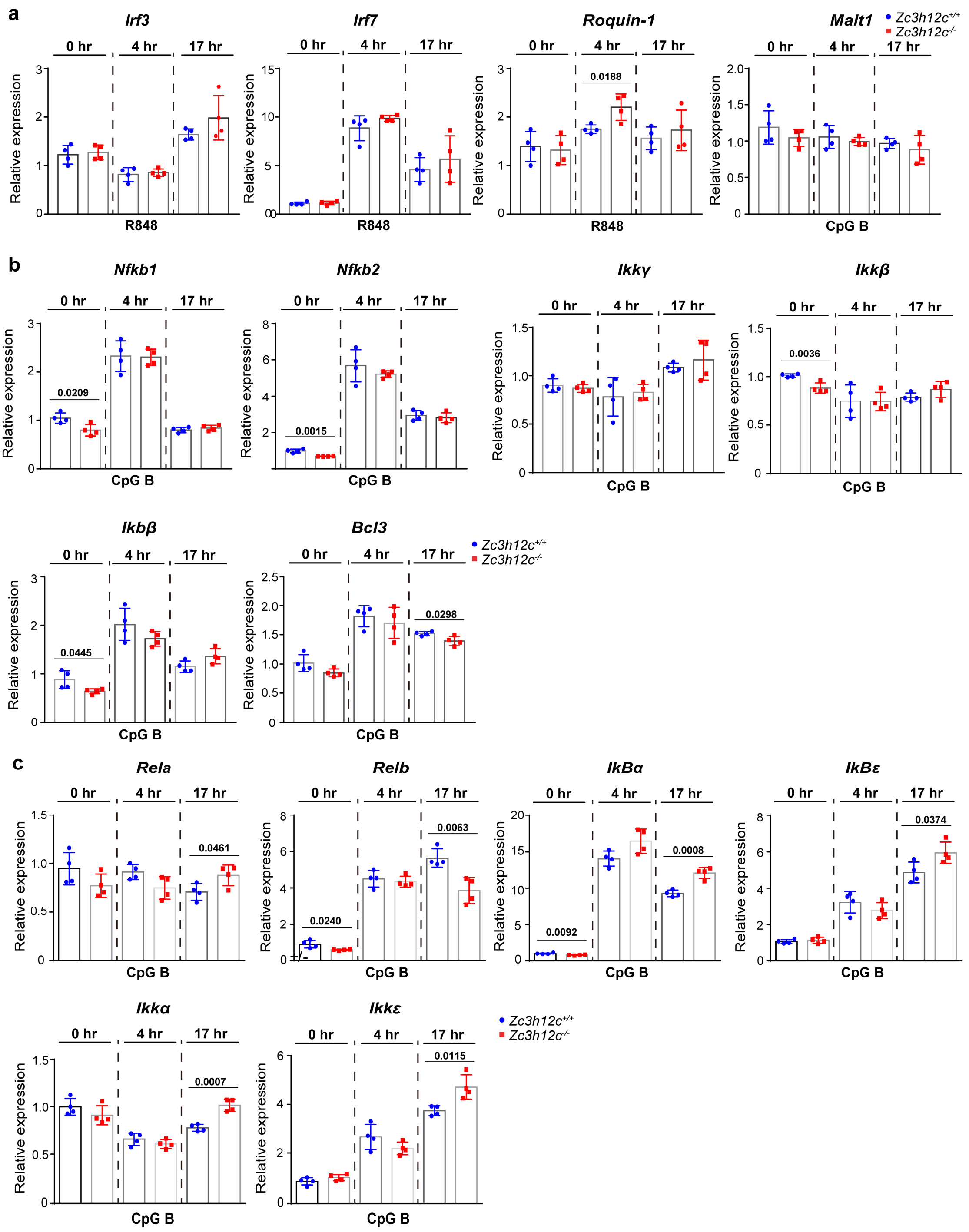

Supplementary Fig. 7 Screening of transcriptional regulators in MCPIP3-deficient pDCs

a-c mRNA expressions of the indicated genes were measured by qPCR in sorted *Zc3h12c*$^{+/+}$/*Zc3h12c*$^{-/-}$ Flt3-pDCs at indicated timepoints after R848 or CpG activation (n=4 mice). Source data are provided as a Source Data file (two-tailed Student's t-test). Data are representative of at least two independent experiments (mean±S.D.)

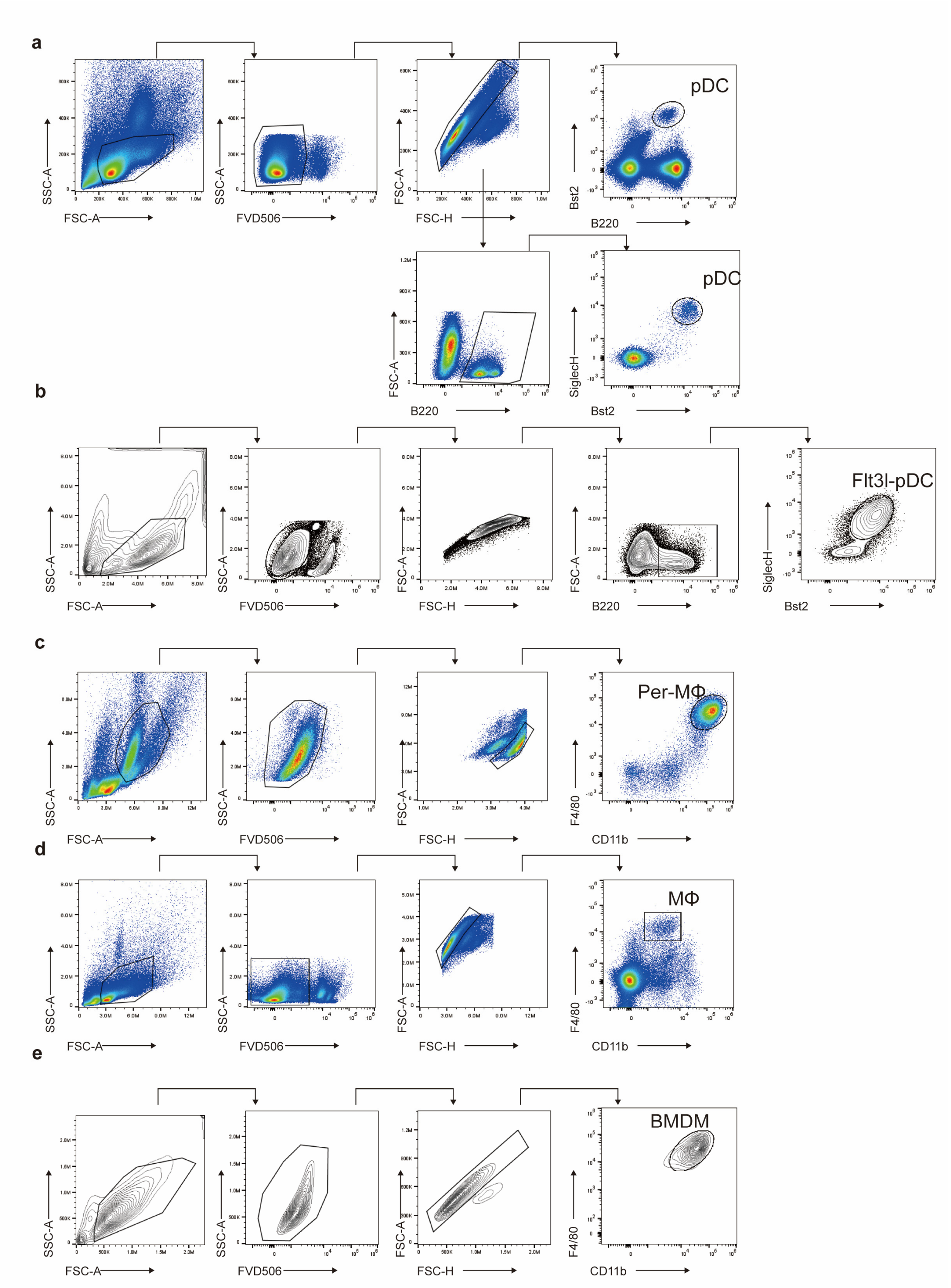

Supplementary Fig. 8 Flow cytometry gating strategies

All immune cells are gated with FSC-A/SSC-A for a live gate. Then a viability dye (FVD506) is used to exclude dead cells. Next, FSC-A/FSC-H is used to exclude doublets. After these gating, cells are specifically gated accordingly below:

a. Gating strategy (B220$^+$ Bst2$^+$) to identify pDC presented on Fig. 3g and Supplementary Fig. 1e; Fig. 2e; Fig. 2g; Fig. 4e-f. Gating strategies (B220$^+$ Bst2$^+$ SiglecH$^+$) to identify pDCs presented on Fig 1f-g, Fig. 3c, e, h; Fig. 6e; Fig. 8e; Supplementary Fig. 1e, h, i.

b. Gating strategies (B220$^+$ Bst2$^+$ SiglecH$^+$) to Flt3l-pDCs presented on Fig. 1f; Fig. 3b, d, I; Fig. 4i; Fig. 6a-d, f, h; Fig. 8f, h-j; Fig. 7.a-c; Supplementary Fig. 2i.

c. Gating strategies (CD11b$^+$ F4/80$^+$) to identify or sort peritoneal cavity macrophages presented on Fig. 1f; Fig. 2f; Fig. 3b; Fig. 7h; Supplementary Fig. 1h; Supplementary Fig. 3b.

d. Gating strategies (CD11b$^+$ F4/80$^+$) to identify splenic macrophages presented on Supplementary Fig. 2c.

e. Gating strategies (CD11b$^+$ F4/80$^+$) to identify or sort bone marrow-derived macrophages (BMDMs) presented on Fig. 1h; Fig. 2a-i; Fig. 3.a, c-f, h; Fig.4a-c, l-n; Fig. 6a-h; Fig. 7g-i; Fig. 8a, e; Supplementary Fig. 2d.

Supplementary Table 1:

Species	Gene/name	Sequence (5' to 3')
Murine	mq*Zc3h12a*-Fwd	CAGATATTACCGTGTTTGTGCC
Murine	mq*Zc3h12a*-Rvs	CTCTAGTTCCCGAAGGATGTG
Murine	mq*Zc3h12b*-Fwd	GATCATGGAGAATGCAACGTG
Murine	mq*Zc3h12b*-Rev	GGTTCCCTTGAGCCTTGG
Murine	mq*Zc3h12c*-Fwd	CGTGCCGAGGAATAAAATTGG
Murine	mq*Zc3h12c*-Rvs	AGGGCGGGACTGTTCTTTTC
Murine	mq*Zc3h12d*-Fwd	TGGCAGCAATGTGGCTAT
Murine	mq*Zc3h12d*-Rvs	GTAGGTGTGTCCTCTGTCTCTA
Murine	mq*Il12p40*-Fwd	TGGTTTGCCATCGTTTTGCTG
Murine	mq*Il12p40*-Rvs	ACAGGTGAGGTTCACTGTTTCT
Murine	mq*A20*-Fwd	ACAGGACTTTGCTACGACAC

Murine	mq*A20*-Rvs	CTGAGGATGTTGCTGAGGAC
Murine	mq*Zfp36*-Fwd	CCGAATCCCTCGGAGGACTT
Murine	mq *Zfp36*-Rvs	GAGCCAAAGGTGCAAAACCA
Murine	mq*Tia1*-Fwd	CACACAGCGTTCACAAGATCA
Murine	mq*Tia1*-Rvs	GGTAGCCATGTCTTTTACCACA
Murine	mq*HuR*-Fwd	GGATGACATTGGGAGAACGAAT
Murine	mq*HuR*-Rvs	TGTCCTGCTACTTTATCCCGAA
Murine	mq*IκBα*-Fwd	CGAGACTTTCGAGGAAATACCC
Murine	mq*IκBα*-Rvs	GTCTGCGTCAAGACTGCTACA
Murine	mq*IκBβ*-Fwd	GCGGATGCCGATGAATGGT
Murine	mq*IκBβ*-Rvs	TGACGTAGCCAAAGACTAAGGG
Murine	mq*IκBε*-Fwd	GCGGAGGCTGAATCACCAG

Murine	mq*IκBε*-Rvs	GAAAGCCCGAACGTGTTCTCA
Murine	mq*Bcl3*-Fwd	CCGGAGGCCCTTTACTACCA
Murine	mq*Bcl3*-Rvs	GGAGTAGGGGTGAGTAGGCAG
Murine	mq*Map3k3(Mekk3)*-Fwd	ATAAGGACACAGGTCACCCAA
Murine	mq*Map3k3(Mekk3)*-Rvs	TGCTCCACATCTTCGTATCTCA
Murine	mq*Prkcz(Pkcζ)*-Fwd	TTACGCCATGAAGGTGGTAAAG
Murine	mq*Prkcz(Pkcζ)*-Rvs	CGCCATTGACATACTCGATGA
Murine	mq*Tnfrsf1a(Tnfr1)*-Fwd	ACAATCCCCTGTAAGGAGACTC
Murine	mq*Tnfrsf1a(Tnfr1)*-Rvs	GCAAGGGACGCACTCACTT
Murine	mq*Nfkbiz*-Fwd	GCTCCGACTCCTCCGATTTC
Murine	mq*Nfkbiz*-Rvs	GAGTTCTTCACGCGAACACC
Murine	mq*Malt1*-Fwd	GGACAAAGTCGCCCTTTTGAT

Murine	mq*Malt1*-Rvs	TCCACAGCGTTACACATCTCA
Murine	mq*Rc3h1(Roquin-1)*-Fwd	AGTGTTCTGAGTCGCCCAATG
Murine	mq*Rc3h1(Roquin-1)*-Rvs	GCGTTCACCTAAAGATCGAGC
Murine	mq*Ltβ*-Fwd	GATGACAGCAAACCGTCGTG
Murine	mq*Ltβ*-Rvs	CCTGGAAGCATTGGATCTCTG
Murine	mq*Il6*-Fwd	CTGCAAGAGACTTCCATCCAG
Murine	mq*Il6*-Rvs	AGTGGTATAGACAGGTCTGTTGG
Murine	mq*Tnf*-Fwd	TAGCCCACGTCGTAGCAAA
Murine	mq*Tnf*-Rvs	GATAGCAAATCGGCTGACG
Murine	mq*Irf7*-Fwd	TCCAGTTGATCCGCATAAGGT
Murine	Murine mq*Irf7*-Rvs	CTTCCCTATTTTCCGTGGCTG
Murine	Murine mq*Irf3*-Fwd	GCGGGACTTCGTACATCTGG
Murine	Murine mq*Irf3*-Rvs	TTCGGTAGGTTTTCCTGGGAG

Murine	Murine mq*Rela* (p65)-Fwd	AGGCTTCTGGGCCTTATGTG
Murine	Murine mq*Rela* (p65)-Rvs	TGCTTCTCTCGCCAGGAATAC
Murine	Murine mq*Relb*-Fwd	CACCGGGTACACCCACATAG
Murine	Murine mq*Relb*-Rvs	ATGCCCAGGTTGTTAAAGCTG
Murine	Murine mq*Rel(C-rel)*-Fwd	TGTCAGCGACTTGAGTGCAT
Murine	Murine mq*Rel(C-rel)*-Rvs	TGACATGGATGGCGTTTCCA
Murine	Murine mq*Nfkb1(p105/p50)*-Fwd	AGAGGGGATTTCGATTCCGC
Murine	Murine mq*Nfkb1(p105/p50)*-Rvs	CCTGTGGGTAGGATTTCTTGTTC
Murine	Murine mq *Nfkb2 (p100/p52)*-Fwd	GGCCGGAAGACCTATCCTACT
Murine	Murine mq *Nfkb2 (p102/p52)*-Rvs	CTACAGACACAGCGCACACT
Murine	Murine mq*Chuk(IKKα)*-Fwd	GTCAGGACCGTGTTCTCAAGG
Murine	Murine mq*Chuk(IKKα)*-Rvs	GCTTCTTTGATGTTACTGAGGGC

Murine	Murine mq*Ikbkb(IKKβ)*-Fwd	AAGTACACCGTGACCGTTGAC
Murine	Murine mq*Ikbkb(IKKβ)*-Rvs	GCTGCCAGTTAGGGAGGAA
Murine	Murine mq*NEMO(IKKγ)*-Fwd	AAGCACCCCTGGAAGAACC
Murine	Murine mq*NEMO(IKKγ)*-Rvs	TCTCAGGAGTACCCTGCTCTG
Murine	Murine mq*Ikbke(IKKi)*-Fwd	CACGTTCGGGCTTTCTGAAGA
Murine	Murine mq*Ikbke(IKKi)*-Rvs	GACAGCTTATAGATGCTCTGCC
Murine	mq*ActB*-Fwd	GTGACGTTGACATCCGTAAAGA
Murine	mq*ActB*-Rvs	GCCGGACTCATCGTACTCC
Human	*ZC3H12C* Forward Primer	GGT GGC GGC TCC CAG GAA TA
Human	*ZC3H12C* Reverse Primer	ACG CCT CTT TTT CAT CCT TCC CCA
Human	*18S* Forward Primer	GTA ACC CGT TGA ACC CCA TT
Human	*18S* Reverse Primer	CCA TCC AAT CGG TAG TAG CG

Virus	LCMV NP1-Fwd	GAGGCTTTCCATCCCAACTAT
Virus	LCMV NP1-Rvs	AAGCTGAAGGCCAAGATCAT
Virus	LCMV NP2-Fwd	CAGAAATGTTGATGCTGGACTGC
Virus	LCMV NP2-Rvs	CAGACCTTGGCTTGCTTTACACAG
Virus	LCMV GP-Fwd	CATTCACCTGGACTTTGTCAGACTC
Virus	LCMV GP-Rvs	GCAACTGCTGTGTTCCCGAAAC
