# Supplementary material for: The RNase MCPIP3 promotes skin inflammation by orchestrating myeloid cytokine response
Source: Nat Commun. 2021 Jul 2;12:4105. doi: 10.1038/s41467-021-24352-w (PMC8253787; doi:10.1038/s41467-021-24352-w)
Supplement: Supplementary file 2 — Reporting Summary [file 41467_2021_24352_MOESM2_ESM.pdf]

nature research

Corresponding author(s): Cliff Y Yang

Last updated by author(s): Apr 18, 2021

Reporting Summary

Nature Research wishes to improve the reproducibility of the work that we publish. This form provides structure for consistency and transparency in reporting. For further information on Nature Research policies, see our Editorial Policies and the Editorial Policy Checklist.

Statistics

For all statistical analyses, confirm that the following items are present in the figure legend, table legend, main text, or Methods section.

n/a	Confirmed	
☐	☒	The exact sample size (*n*) for each experimental group/condition, given as a discrete number and unit of measurement
☐	☒	A statement on whether measurements were taken from distinct samples or whether the same sample was measured repeatedly
☐	☒	The statistical test(s) used AND whether they are one- or two-sided<br>*Only common tests should be described solely by name; describe more complex techniques in the Methods section.*
☐	☒	A description of all covariates tested
☐	☒	A description of any assumptions or corrections, such as tests of normality and adjustment for multiple comparisons
☐	☒	A full description of the statistical parameters including central tendency (e.g. means) or other basic estimates (e.g. regression coefficient) AND variation (e.g. standard deviation) or associated estimates of uncertainty (e.g. confidence intervals)
☐	☒	For null hypothesis testing, the test statistic (e.g. *F*, *t*, *r*) with confidence intervals, effect sizes, degrees of freedom and *P* value noted<br>*Give P values as exact values whenever suitable.*
☒	☐	For Bayesian analysis, information on the choice of priors and Markov chain Monte Carlo settings
☒	☐	For hierarchical and complex designs, identification of the appropriate level for tests and full reporting of outcomes
☒	☐	Estimates of effect sizes (e.g. Cohen's *d*, Pearson's *r*), indicating how they were calculated

Our web collection on statistics for biologists contains articles on many of the points above.

Software and code

Policy information about availability of computer code

Data collection	FACSDiva v8.0.1, StepOne v2.1. For additional details please refer to Methods.
Data analysis	FlowJo vX; Prism v6; Excel v14.3.0; ImageJ 2. For additional details please refer to Methods.

For manuscripts utilizing custom algorithms or software that are central to the research but not yet described in published literature, software must be made available to editors and reviewers. We strongly encourage code deposition in a community repository (e.g. GitHub). See the Nature Research guidelines for submitting code & software for further information.

Data

Policy information about availability of data

All manuscripts must include a data availability statement. This statement should provide the following information, where applicable:
- Accession codes, unique identifiers, or web links for publicly available datasets
- A list of figures that have associated raw data
- A description of any restrictions on data availability

No novel datasets are generated in this study. Data that support the findings of this study are re-analysis of publicly available datasets. Patient biopsy datasets are available at Gene Expression Omnibus (GEO) repository (https://www.ncbi.nlm.nih.gov/gdsin) with accession codes of GDS3539; GDS4600; GSE53552. RNA-seq datasets are available at The Immunological Genome Project (ImmGen) (https://www.immgen.org) with the search term “Zc3h12c”. SNP analysis is available at The Genotype-Tissue Expression (GTEx) (https://www.gtexportal.org/) at with the search term of “rs4561177”. Source data are provided with this paper.

Field-specific reporting

Please select the one below that is the best fit for your research. If you are not sure, read the appropriate sections before making your selection.

[x] Life sciences [ ] Behavioural & social sciences [ ] Ecological, evolutionary & environmental sciences

For a reference copy of the document with all sections, see nature.com/documents/nr-reporting-summary-flat.pdf

Life sciences study design

All studies must disclose on these points even when the disclosure is negative.

Sample size	No statistical methods were used to predetermine sample size. Sample size were chosen based on the maximum number of mice available at the moment of experiments. Since the mice are on identical genetic backgrounds, a minimum of 3 individual mice were commonly recognized in the field to sufficiently detect differences between genotypes or conditions.
Data exclusions	Human samples were known to vary greatly, and extreme outliers would impact analysis of a small dataset with a smaller sample size. Thus, a exclusion criteria is pre-determined with a commonly used value of Z-scores. Z-score is used to mathematically find outliers and is generally set at a threshold of 3 or -3. In Figure 1e, two human samples (one each from CD4+ T and macrophages groups) were excluded as outliers, due to their Z-scores exceeding ± 3.
Replication	Experiments were replicated successfully for least twice as described throughout the paper and in the Methods.
Randomization	Mice of similar ages and sex were used for all the experiments reported. Samples were randomly assigned.
Blinding	For mice IMQ and IL-23 induced psoriasis, mPASI and ear thickness measurements were performed blinded, without knowledge of mice genotype. For other experiments, the investigators were not blinded to the identities of the samples because treatments and data collection were performed by the same people. During data analysis, investigators were not blinded to group allocation, as this is performed by the same people. The reason for these unblindings is due to a lack of personnel and resources, as investigators have to collect and analysis data by themselves. For human studies, there was no blinding in this study as we did not have different treatment/experimental groups and therefore was not relevant.

Reporting for specific materials, systems and methods

We require information from authors about some types of materials, experimental systems and methods used in many studies. Here, indicate whether each material, system or method listed is relevant to your study. If you are not sure if a list item applies to your research, read the appropriate section before selecting a response.

Materials & experimental systems

n/a	Involved in the study	
[ ]	[x]	Antibodies
[ ]	[x]	Eukaryotic cell lines
[x]	[ ]	Palaeontology and archaeology
[ ]	[x]	Animals and other organisms
[ ]	[x]	Human research participants
[x]	[ ]	Clinical data
[x]	[ ]	Dual use research of concern

Methods

n/a	Involved in the study	
[x]	[ ]	ChIP-seq
[ ]	[x]	Flow cytometry
[x]	[ ]	MRI-based neuroimaging

Antibodies

Antibodies used

For flow cytometry ,
Company: eBioscience CD45 FITC Clone: 30-F11 Catalogue #: 11-0451-82
Company: eBioscience CD45 PE Clone: 30-F11 Catalogue #: 12-0451-82
Company: eBioscience CD45 APC Clone: 30-F11 Catalogue #: 17-0451-82
Company: eBioscience CD45.1 PE-Cyanine7 Clone: A20 Catalogue #: 25-0453-82
Company: eBioscience CD45.1 PB Clone: A20 Catalogue #: 48-0453-82
Company: eBioscience CD45.2 APC-Cyanine7 Clone: 104 Catalogue #: 47-0454-82
Company: Biolegend XCR1 PerCP/Cyanine5.5 Clone: ZET Catalogue #: 148207
Company: eBioscience CD172a PE Clone: P84 Catalogue #: 12-1721-82
Company: eBioscience CD172a PE-Cyanine7 Clone: P84 Catalogue #: 25-1721-82
Company: eBioscience MHC Class II PE Clone: M5/114.15.2 Catalogue #: 12-5321-82
Company: eBioscience MHC Class II PE-Cyanine7 Clone: M5/114.15.2 Catalogue #: 25-5321-82
Company: eBioscience MHC Class II PB Clone: M5/114.15.2 Catalogue #: 48-5321-82
Company: eBioscience CD11c PE Clone: N418 Catalogue #: 12-0114-82

Company: eBioscience CD11c APC-Cyanine7 Clone: N418 Catalogue #: 47-0114-82
Company: eBioscience CD8a PerCP/Cyanine5.5 Clone: 53-6.7 Catalogue #: 35-0081-82
Company: eBioscience CD11b PerCP-Cyanine5.5 Clone: M1/70 Catalogue #: 45-0112-82
Company: eBioscience CD11b FITC Clone: M1/70 Catalogue #: 11-0112-82
Company: eBioscience CD4 APC-Cyanine7 Clone: RM4-5 Catalogue #: 47-0042-82
Company: eBioscience CD4 eFluor 450 Clone: RM4-5 Catalogue #: 48-0042-82
Company: eBioscience BST2 APC Clone: eBio927 Catalogue #: 17-3172-82
Company: eBioscience BST2 FITC Clone: eBio927 Catalogue #: 11-3172-82
Company: eBioscience SIGLEC H APC Clone: eBio440c Catalogue #: 17-0333-82
Company: eBioscience SIGLEC H FITC Clone: eBio440c Catalogue #: 11-0333-82
Company: eBioscience B220 eFluor 450 Clone: RA3-6B2 Catalogue #: 48-0452-82
Company: eBioscience TNF alpha PE-Cyanine7 Clone: MP6-XT22 Catalogue #: 25-7321-82
Company: eBioscience CD24 PE Clone: M1/69 Catalogue #: 12-0242-82
Company: eBioscience F4/80 PE Clone: BM8 Catalogue #: 12-4801-82
Company: eBioscience F4/80 eFluor 450 Clone: BM8 Catalogue #: MF48004-3
Company: eBioscience IgD APC-eFluor 780 Clone: 11-26c (11-26) Catalogue #: 47-5993-82
Company: eBioscience IgM PE Clone: II/41 Catalogue #: 12-5790-82
Company: eBioscience IgM Super Bright 600 Clone: II/41 Catalogue #: 63-5790-82
Company: eBioscience CD93 APC Clone: AA4.1 Catalogue #: 17-5892-82
Company: Biolegend CD5 APC Clone: 53-7.3 Catalogue #: 100626
Company: Biolegend Ly-6C Alexa Fluor® 488 Clone: HK1.4 Catalogue #: 128022
Company: eBioscience Ly-6G eFluor 450 Clone: 1A8-Ly6g Catalogue #: 48-9668-82
Company: eBioscience CD3e APC Clone: 145-2C11 Catalogue #: MA1-10186
Company: eBioscience CD3e eFluor 450 Clone: 145-2C11 Catalogue #: 48-0031-82
Company: eBioscience TCR-γδ APC Clone: eBioGL3 (GL-3, GL3) Catalogue #: 17-5711-82
Company: eBioscience TCR-γδ PE-Cyanine7 Clone: eBioGL3 (GL-3, GL3) Catalogue #: 25-5711-82
Company: eBioscience TCR β PE Clone: H57-597 Catalogue #: 12-5961-82
Company: eBioscience TCR β APC-eFluor 780 Clone: H57-597 Catalogue #: 47-5961-82
Company: eBioscience IFN-γ PE Clone: XMG1.2 Catalogue #: 12-7311-82
Company: eBioscience IFN-γ PerCP/Cyanine5.5 Clone: XMG1.2 Catalogue #: 45-7311-82
Company: eBioscience IL-17A FITC Clone: eBio17B7 Catalogue #: 11-7177-81
Company: Biolegend CD80 FITC Clone: 16-10A1 Catalogue #: 104706
Company: Biolegend CD80 PE Clone: 16-10A1 Catalogue #: 104708
Company: MBL Ly49Q PE Clone: 2E/6 Catalogue #: D160-4
Company: eBioscience CD86 FITC Clone: GL1 Catalogue #: 11-0862-82
Company: eBioscience CD86 PE Clone: GL1 Catalogue #: 12-0862-82
Company: eBioscience CD199 PerCP-eFluor 710 Clone: eBioCW-1.2 (CW-1.2) Catalogue #: 46-1991-82
Company: eBioscience CD64 PerCP-eFluor 710 Clone: X54-5/7.1 Catalogue #: 46-0641-82
Company: pblassay IFNa FITC Clone: RMMA-1 Catalogue #: 22100-3
Company: Biolegend CCR2 PE-Cyanine7 Clone: QA18A56 Catalogue #: 160108
Company: eBioscience Ly-6A/E (Sca-1) APC Clone: D7 Catalogue #: 17-5981-82
Company: eBioscience Ly-6A/E (Sca-1) PE-Cyanine7 Clone: D7 Catalogue #: 25-5981-82
Company: eBioscience IL-12p40 eFluor 660 Clone: C17.8 Catalogue #: 50-7123-82
Company: invivogen IL-6 N.A Clone: 10F9 Catalogue #: mabg-mil6-3
Company: Sigma-Aldrich anti-Flag (M2) -HRP N.A Clone: A8592 Catalogue #: A8592-.2MG
Company: Sigma-Aldrich anti-c-Myc-HRP N.A Clone: 4A6 Catalogue #: 16-213
Company: Sigma-Aldrich anti-β-actin-HRP N.A Clone: A3854 Catalogue #: A3854-200UL

For western blotting, mouse monoclonal anti-Flag (M2) peroxidase (HRP)(Sigma-Aldrich, Cat#A8592), Mouse monoclonal anti-c-Myc-HRP (Roche Applied Science, Cat#11814150001), Mouse monoclonal anti-β-actin (Sigma-Aldrich, Cat# A3854).

Validation

All antibodies used were commercially available and validated by corresponding manufacturers. The following companies have general validation/reproducibility statements. BD (https://www.bdbiosciences.com/us/go/reproducibility/overview). Biolegend (https://www.biolegend.com/en-us/reproducibility). Roche applied science (https://lifescience.roche.com/en_cn/tools-and-resources/documents.html). Sigma aldrich(https://www.sigmaaldrich.com/technical-documents/articles/biology/antibody-standard-validation.html)
Company: eBioscience CD45 FITC Clone: 30-F11 Catalogue #: 11-0451-82 https://www.thermofisher.com/cn/zh/antibody/product/CD45-Antibody-clone-30-F11-Monoclonal/11-0451-82

Company: eBioscience CD45 PE Clone: 30-F11 Catalogue #: 12-0451-82 https://www.thermofisher.com/cn/zh/antibody/product/CD45-Antibody-clone-30-F11-Monoclonal/12-0451-82

Company: eBioscience CD45 APC Clone: 30-F11 Catalogue #: 17-0451-82 https://www.thermofisher.com/cn/zh/antibody/product/CD45-Antibody-clone-30-F11-Monoclonal/17-0451-82

Company: eBioscience CD45.1 PE-Cyanine7 Clone: A20 Catalogue #: 25-0453-82 https://www.thermofisher.com/cn/zh/antibody/product/CD45-1-Antibody-clone-A20-Monoclonal/25-0453-82

Company: eBioscience CD45.1 PB Clone: A20 Catalogue #: 48-0453-82 https://www.thermofisher.com/cn/zh/antibody/product/

CD45-1-Antibody-clone-A20-Monoclonal/48-0453-82

Company: eBioscience CD45.2 APC-Cyanine7 Clone: 104 Catalogue #: 47-0454-82 https://www.thermofisher.com/cn/zh/antibody/product/CD45-2-Antibody-clone-104-Monoclonal/47-0454-82

Company: Biolegend XCR1 PerCP/Cyanine5.5 Clone: ZET Catalogue #: 148207 https://www.biolegend.com/en-us/products/percp-cyanine5-5-anti-mouse-rat-xcr1-antibody-10397

Company: eBioscience CD172a PE Clone: P84 Catalogue #: 12-1721-82 https://www.thermofisher.com/cn/zh/antibody/product/CD172a-SIRP-alpha-Antibody-clone-P84-Monoclonal/12-1721-82?imageId=89824

Company: eBioscience CD172a PE-Cyanine7 Clone: P84 Catalogue #: 25-1721-82 https://www.thermofisher.com/cn/zh/antibody/product/CD172a-SIRP-alpha-Antibody-clone-P84-Monoclonal/25-1721-82?imageId=309805

Company: eBioscience MHC Class II PE Clone: M5/114.15.2 Catalogue #: 12-5321-82 https://www.thermofisher.com/cn/zh/antibody/product/MHC-Class-II-I-A-I-E-Antibody-clone-M5-114-15-2-Monoclonal/12-5321-82?imageId=89900

Company: eBioscience MHC Class II PE-Cyanine7 Clone: M5/114.15.2 Catalogue #: 25-5321-82 https://www.thermofisher.com/cn/zh/antibody/product/MHC-Class-II-I-A-I-E-Antibody-clone-M5-114-15-2-Monoclonal/25-5321-82?imageId=91425

Company: eBioscience MHC Class II PB Clone: M5/114.15.2 Catalogue #: 48-5321-82 https://www.thermofisher.com/cn/zh/antibody/product/MHC-Class-II-I-A-I-E-Antibody-clone-M5-114-15-2-Monoclonal/48-5321-82?imageId=92211

Company: eBioscience CD11c PE Clone: N418 Catalogue #: 12-0114-82 https://www.thermofisher.com/cn/zh/antibody/product/CD11c-Antibody-clone-N418-Monoclonal/12-0114-82?imageId=89429

Company: eBioscience CD11c APC-Cyanine7 Clone: N418 Catalogue #: 47-0114-82 https://www.thermofisher.com/cn/zh/antibody/product/CD11c-Antibody-clone-N418-Monoclonal/47-0114-82?imageId=92019

Company: eBioscience CD8a PerCP/Cyanine5.5 Clone: 53-6.7 Catalogue #: 35-0081-82 https://www.thermofisher.com/cn/zh/antibody/product/CD8a-Antibody-clone-53-6-7-Monoclonal/35-0081-82?imageId=91539

Company: eBioscience CD11b PerCP-Cyanine5.5 Clone: M1/70 Catalogue #: 45-0112-82 https://www.thermofisher.com/cn/zh/antibody/product/CD11b-Antibody-clone-M1-70-Monoclonal/45-0112-82?imageId=91627

Company: eBioscience CD11b FITC Clone: M1/70 Catalogue #: 11-0112-82 https://www.thermofisher.com/cn/zh/antibody/product/CD11b-Antibody-clone-M1-70-Monoclonal/11-0112-82?imageId=121108

Company: eBioscience CD4 APC-Cyanine7 Clone: RM4-5 Catalogue #: 47-0042-82 https://www.thermofisher.com/cn/zh/antibody/product/CD4-Antibody-clone-RM4-5-Monoclonal/47-0042-82?imageId=92007

Company: eBioscience CD4 eFluor 450 Clone: RM4-5 Catalogue #: 48-0042-82 https://www.thermofisher.com/cn/zh/antibody/product/CD4-Antibody-clone-RM4-5-Monoclonal/48-0042-82?imageId=92103

Company: eBioscience BST2 APC Clone: eBio927 Catalogue #: 17-3172-82 https://www.thermofisher.com/cn/zh/antibody/product/CD317-BST2-PDCA-1-Antibody-clone-eBio927-Monoclonal/17-3172-82?imageId=91122

Company: eBioscience BST2 FITC Clone: eBio927 Catalogue #: 11-3172-82 https://www.thermofisher.com/cn/zh/antibody/product/CD317-BST2-PDCA-1-Antibody-clone-eBio927-Monoclonal/11-3172-82?imageId=89669

Company: eBioscience SIGLEC H APC Clone: eBio440c Catalogue #: 17-0333-82 https://www.thermofisher.com/cn/zh/antibody/product/SIGLEC-H-Antibody-clone-eBio440c-Monoclonal/17-0333-82?imageId=492489

Company: eBioscience SIGLEC H FITC Clone: eBio440c Catalogue #: 11-0333-82 https://www.thermofisher.com/cn/zh/antibody/product/SIGLEC-H-Antibody-clone-eBio440c-Monoclonal/11-0333-82?imageId=89574

Company: eBioscience B220 eFluor 450 Clone: RA3-6B2 Catalogue #: 48-0452-82 https://www.thermofisher.com/cn/zh/antibody/product/CD45R-B220-Antibody-clone-RA3-6B2-Monoclonal/48-0452-82

Company: eBioscience TNF alpha PE-Cyanine7 Clone: MP6-XT22 Catalogue #: 25-7321-82 https://www.thermofisher.com/cn/zh/antibody/product/TNF-alpha-Antibody-clone-MP6-XT22-Monoclonal/25-7321-82?imageId=91487

Company: eBioscience CD24 PE Clone: M1/69 Catalogue #: 12-0242-82 https://www.thermofisher.com/cn/zh/antibody/product/CD24-Antibody-clone-M1-69-Monoclonal/12-0242-82

Company: eBioscience F4/80 PE Clone: BM8 Catalogue #: 12-4801-82 https://www.thermofisher.com/cn/zh/antibody/product/F4-80-Antibody-clone-BM8-Monoclonal/12-4801-82?imageId=89880

Company: eBioscience F4/80 eFluor 450 Clone: BM8 Catalogue #: MF48004-3 https://www.thermofisher.com/cn/zh/antibody/product/F4-80-Antibody-clone-BM8-Monoclonal/MF48004-3?imageId=2520

Company: eBioscience IgD APC-eFluor 780 Clone: 11-26c (11-26) Catalogue #: 47-5993-82 https://www.thermofisher.com/cn/zh/antibody/product/IgD-Antibody-clone-11-26c-11-26-Monoclonal/47-5993-82

Company: eBioscience IgM PE Clone: II/41 Catalogue #: 12-5790-82 https://www.thermofisher.com/cn/zh/antibody/product/IgM-Antibody-clone-II-41-Monoclonal/12-5790-82?imageId=89917

Company: eBioscience IgM Super Bright 600 Clone: II/41 Catalogue #: 63-5790-82 https://www.thermofisher.com/cn/zh/antibody/product/IgM-Antibody-clone-II-41-Monoclonal/63-5790-82?imageId=153403

Company: eBioscience CD93 APC Clone: AA4.1 Catalogue #: 17-5892-82 https://www.thermofisher.com/cn/zh/antibody/product/CD93-AA4-1-Antibody-clone-AA4-1-Monoclonal/17-5892-82

Company: Biolegend CD5 APC Clone: 53-7.3 Catalogue #: 100626 https://www.biolegend.com/en-us/products/apc-anti-mouse-cd5-antibody-9923

Company: Biolegend Ly-6C Alexa Fluor® 488 Clone: HK1.4 Catalogue #: 128022 https://www.biolegend.com/en-us/products/alexa-fluor-488-anti-mouse-ly-6c-antibody-6756

Company: eBioscience Ly-6G eFluor 450 Clone: 1A8-Ly6g Catalogue #: 48-9668-82 https://www.thermofisher.com/cn/zh/antibody/product/Ly-6G-Antibody-clone-1A8-Ly6g-Monoclonal/48-9668-82?imageId=93074

Company: eBioscience CD3e APC Clone: 145-2C11 Catalogue #: MA1-10186 https://www.thermofisher.com/cn/zh/antibody/product/CD3e-Antibody-clone-145-2C11-Monoclonal/MA1-10186

Company: eBioscience CD3e eFluor 450 Clone: 145-2C11 Catalogue #: 48-0031-82 https://www.thermofisher.com/cn/zh/antibody/product/CD3e-Antibody-clone-145-2C11-Monoclonal/48-0031-82?imageId=121475

Company: eBioscience TCR-γδ APC Clone: eBioGL3 (GL-3, GL3) Catalogue #: 17-5711-82 https://www.thermofisher.com/cn/zh/antibody/product/TCR-gamma-delta-Antibody-clone-eBioGL3-GL-3-GL3-Monoclonal/17-5711-82?imageId=91146

Company: eBioscience TCR-γδ PE-Cyanine7 Clone: eBioGL3 (GL-3, GL3) Catalogue #: 25-5711-82 https://www.thermofisher.com/cn/zh/antibody/product/TCR-gamma-delta-Antibody-clone-eBioGL3-GL-3-GL3-Monoclonal/25-5711-82?imageId=91430

Company: eBioscience TCR β PE Clone: H57-597 Catalogue #: 12-5961-82 https://www.thermofisher.com/cn/zh/antibody/product/TCR-beta-Antibody-clone-H57-597-Monoclonal/12-5961-82?imageId=89968

Company: eBioscience TCR β APC-eFluor 780 Clone: H57-597 Catalogue #: 47-5961-82 https://www.thermofisher.com/cn/zh/antibody/product/TCR-beta-Antibody-clone-H57-597-Monoclonal/47-5961-82?imageId=92087

Company: eBioscience IFN-γ PE Clone: XMG1.2 Catalogue #: 12-7311-82 https://www.thermofisher.com/cn/zh/antibody/product/IFN-gamma-Antibody-clone-XMG1-2-Monoclonal/12-7311-82?imageId=120906

Company: eBioscience IFN-γ PerCP/Cyanine5.5 Clone: XMG1.2 Catalogue #: 45-7311-82 https://www.thermofisher.com/cn/zh/antibody/product/IFN-gamma-Antibody-clone-XMG1-2-Monoclonal/45-7311-82?imageId=91675

Company: eBioscience IL-17A FITC Clone: eBio17B7 Catalogue #: 11-7177-81 https://www.thermofisher.com/cn/zh/antibody/product/IL-17A-Antibody-clone-eBio17B7-Monoclonal/11-7177-81?imageId=89349

Company: Biolegend CD80 FITC Clone: 16-10A1 Catalogue #: 104706 https://www.biolegend.com/en-us/products/fitc-anti-mouse-cd80-antibody-41

Company: Biolegend CD80 PE Clone: 16-10A1 Catalogue #: 104708 https://www.biolegend.com/en-us/products/pe-anti-mouse-cd80-antibody-43

Company: MBL Ly49Q PE Clone: 2E/6 Catalogue #: D140-4 https://ruo.mbl.co.jp/d140-4

Company: eBioscience CD86 FITC Clone: GL1 Catalogue #: 11-0862-82 https://www.thermofisher.com/cn/zh/antibody/product/CD86-B7-2-Antibody-clone-GL1-Monoclonal/11-0862-82?imageId=121134

Company: eBioscience CD86 PE Clone: GL1 Catalogue #: 12-0862-82 https://www.thermofisher.com/cn/zh/antibody/product/CD86-B7-2-Antibody-clone-GL1-Monoclonal/12-0862-82?imageId=120826

Company: eBioscience CD199 PerCP-eFluor 710 Clone: eBioCW-1.2 (CW-1.2) Catalogue #: 46-1991-82 https://www.thermofisher.com/cn/zh/antibody/product/CD199-CCR9-Antibody-clone-eBioCW-1-2-CW-1-2-Monoclonal/46-1991-82

Company: eBioscience CD64 PerCP-eFluor 710 Clone: X54-5/7.1 Catalogue #: 46-0641-82 https://www.thermofisher.com/cn/zh/antibody/product/CD64-Antibody-clone-X54-5-7-1-Monoclonal/46-0641-82?imageId=258087

Company: pblassay IFNa FITC Clone: RMMA-1 Catalogue #: 22100-3 https://www.pblassaysci.com/antibodies/fitc-conjugated-anti-mouse-ifn-alpha-antibody-clone-rmma-1-mab-221003

Company: Biolegend CCR2 PE-Cyanine7 Clone: QA18A56 Catalogue #: 160108 https://www.biolegend.com/en-us/products/pecyanine7-anti-mouse-cd192-ccr2-recombinant-antibody-20298

Company: eBioscience Ly-6A/E (Sca-1) APC Clone: D7 Catalogue #: 17-5981-82 https://www.thermofisher.com/cn/zh/antibody/product/Ly-6A-E-Sca-1-Antibody-clone-D7-Monoclonal/17-5981-82?imageId=91189

Company: eBioscience Ly-6A/E (Sca-1) PE-Cyanine7 Clone: D7 Catalogue #: 25-5981-82 https://www.thermofisher.com/cn/zh/antibody/product/Ly-6A-E-Sca-1-Antibody-clone-D7-Monoclonal/25-5981-82?imageId=91464

Company: eBioscience IL-12p40 eFluor 660 Clone: C17.8 Catalogue #: 50-7123-82 https://www.thermofisher.com/cn/zh/antibody/product/IL-12-IL-23-p40-Antibody-clone-C17-8-Monoclonal/50-7123-82?imageId=92415

Company: invivogen IL-6 N.A Clone: 10F9 Catalogue #: mabg-mil6-3 https://www.invivogen.com/anti-mil6-igg

Company: Sigma-Aldrich anti-Flag (M2) -HRP N.A Clone: A8592 Catalogue #: A8592-.2MG https://www.sigmaaldrich.cn/CN/zh/product/sigma/a8592?context=product

Company: Sigma-Aldrich anti-c-Myc-HRP N.A Clone: 4A6 Catalogue #: 16-213 https://www.sigmaaldrich.cn/CN/zh/product/mm/16213?context=product

Company: Sigma-Aldrich anti-β-actin-HRP N.A Clone: A3854 Catalogue #: A3854-200UL https://www.sigmaaldrich.cn/CN/zh/product/sigma/a3854?context=product

Eukaryotic cell lines

Policy information about cell lines

Cell line source(s): 293T were purchased from ATCC (CRL-3216), Flt3L-B16 cell line is generated from ATCC-purchased B16 (ATCC, CRL-6475).L929 is purchase from ATCC (CRL-6364).

Authentication: Authentication of 293T ,B16, L929 is provided by ATCC with STR profiling. The complete authentication information is provided at: 293T: https://www.atcc.org/products/all/CRL-6475.aspx; B16: https://www.atcc.org/products/all/CRL-3216.aspx. and L929: https://www.atcc.org/products/all/CRL-6364.aspx.

Mycoplasma contamination: The cell line was confirmed to be negative for mycoplasma contamination by PCR.

Commonly misidentified lines (See ICLAC register): No commonly misidentified cell lines were used.

Animals and other organisms

Policy information about studies involving animals; ARRIVE guidelines recommended for reporting animal research

Laboratory animals: C57BL/6J (WT), B6.SJL-Ptprc¬aPepcb/BoyJ (CD45.1+), B6.129S2-Ifnar1tm1Agt/Mmjax (Ifnar1-/-), B6.129P2-Lyz2tm1(cre)Ifo/J (Lyz2-Cre) and B6.Cg-Tg (Itgax-cre)1-1Reiz/J (Itgax-Cre) were purchased from The Jackson Laboratory. Zc3h12c-/- and Zc3h12cfl/fl mice were generated in-house and could be provided upon reasonable request. All mice used are sex matched, with no bias for female or male, at the age -matched range of 6-12 weeks. The mice are housed in 12 hours/12 hours light-dark cycle, ambient temperature of 18-22 degrees, and 50-60% humidity.

Wild animals: This study did not involve the use of wild animals.

Field-collected samples: The study did not involve samples collected from the field.

Ethics oversight: All mice protocols were followed according to the institutional guidelines and protocols approved by the Animal Ethics Committee of Sun Yat-sen University.

Note that full information on the approval of the study protocol must also be provided in the manuscript.

Human research participants

Policy information about studies involving human research participants

Population characteristics: No covariate-relevant population characteristics were used.

Recruitment: Peripheral blood samples were collected from healthy donors, after taking written informed consent, in accordance with the Declaration of Helsinki and as recommended by the institutional review board of CSIR-Indian Institute of Chemical Biology, Kolkata, India. There is no potential bias in this study as there is no different treatments or groups, all samples are used in the

	analysis.
Ethics oversight	Approved by the institutional review board of CSIR-Indian Institute of Chemical Biology, Kolkata, India.

Note that full information on the approval of the study protocol must also be provided in the manuscript.

Flow Cytometry

Plots

Confirm that:

- [x] The axis labels state the marker and fluorochrome used (e.g. CD4-FITC).
- [x] The axis scales are clearly visible. Include numbers along axes only for bottom left plot of group (a 'group' is an analysis of identical markers).
- [x] All plots are contour plots with outliers or pseudocolor plots.
- [x] A numerical value for number of cells or percentage (with statistics) is provided.

Methodology

Sample preparation	Cells were blocked with Fc blocker(CD16/32), and stained for specific surface markers. For intracellular staining, cells were fixed and permeabilized and stained for intracellular cytokines by fixation/permeabilization kit (eBiosciences).
Instrument	Flow cytometry data were collected by CytoFLEX (Beckman Coulter) and FACSAria II (BD Biosciences) for cell sorting.
Software	BD FACSDiva software (BD Biosciences) for data acquisition and analyzed using FlowJo software version X (Tree Star). Data was graphed using Prism 6 (Graphpad).
Cell population abundance	After sorting we purity was determined to be >95%.
Gating strategy	The gating strategy is shown in Supplementary figure 8. All antibodies were titrated on murine splenic cells to determine optional concentrations for the separation of positive or negative populations. All population markers were on bifurcated markers and clearly defined positive from negative.<br><br>All immune cells are gated with FSC-A/SSC-A for a live gate. Then a viability dye (FVD506) is used to exclude dead cells. Next, FSC-A/FSC-H is used to exclude doublets. After these gating, cells are specifically gated accordingly below:<br><br>a. Gating strategy (B220+ Bst2+) to identify pDC presented on Fig. 3g and Supplementary Fig. 1e; Fig. 2e; Fig. 2g; Fig. 4e-f. Gating strategies (B220+ Bst2+ SiglecH+) to identify pDCs presented on Fig 1f-g, Fig. 3c, e, h; Fig. 6e; Fig. 8e; Supplementary Fig. 1e, h, i.<br><br>b. Gating strategies (B220+ Bst2+ SiglecH+) to Flt3l-pDCs presented on Fig. 1f; Fig. 3b, d, I; Fig. 4i; Fig. 6a-d, f, h; Fig. 8f, h-j; Fig. 7.a-c; Supplementary Fig. 2i.<br><br>c. Gating strategies (CD11b+ F4/80+) to identify or sort peritoneal cavity macrophages presented on Fig. 1f; Fig. 2f; Fig. 3b; Fig. 7h; Supplementary Fig. 1h; Supplementary Fig. 3b.<br><br>d. Gating strategies (CD11b+ F4/80+) to identify splenic macrophages presented on Supplementary Fig. 2c.<br><br>e. Gating strategies (CD11b+ F4/80+) to identify or sort bone marrow-derived macrophages (BMDMs) presented on Fig. 1h; Fig. 2a-i; Fig. 3.a, c-f, h; Fig.4a-c, l-n; Fig. 6a-h; Fig. 7g-i; Fig. 8a, e; Supplementary Fig. 2d.

- [x] Tick this box to confirm that a figure exemplifying the gating strategy is provided in the Supplementary Information.
